# A Computational Approach to Estimate Interorgan Metabolic Transport in a Mammal

**DOI:** 10.1371/journal.pone.0100963

**Published:** 2014-06-27

**Authors:** Xiao Cui, Lars Geffers, Gregor Eichele, Jun Yan

**Affiliations:** 1 Functional Genomics Group, CAS-MPG Partner Institute for Computational Biology, Shanghai, China; 2 Department of Genes and Behavior, Max Planck Institute for Biophysical Chemistry, Göttingen, Germany; University of Hull, United Kingdom

## Abstract

In multicellular organisms metabolism is distributed across different organs, each of which has specific requirements to perform its own specialized task. But different organs also have to support the metabolic homeostasis of the organism as a whole by interorgan metabolite transport. Recent studies have successfully reconstructed global metabolic networks in tissues and cell types and attempts have been made to connect organs with interorgan metabolite transport. Instead of these complicated approaches to reconstruct global metabolic networks, we proposed in this study a novel approach to study interorgan metabolite transport focusing on transport processes mediated by solute carrier (Slc) transporters and their couplings to cognate enzymatic reactions. We developed a computational approach to identify and score potential interorgan metabolite transports based on the integration of metabolism and transports in different organs in the adult mouse from quantitative gene expression data. This allowed us to computationally estimate the connectivity between 17 mouse organs via metabolite transport. Finally, by applying our method to circadian metabolism, we showed that our approach can shed new light on the current understanding of interorgan metabolite transport at a whole-body level in mammals.

## Introduction

Metabolism is a vital process to support normal life in every organism. In mammals, it is comprised of integrated cellular and biochemical processes catalyzed by about 2000 enzymes [1]. The vast repertoire of enzymatic reactions in combination with various metabolite transport systems is required to convert small molecules and other chemical substances into a variety of biomolecules necessary to perform tissue- and cell-type specific functions.

During the past ten years, metabolism research has directed its focus on the systems level, taking advantage of the advances provided by genome-scale studies. The human metabolic reconstruction models Recon 1 and Recon 2 [2], [3] were aimed at building a comprehensive network in order to facilitate studies of metabolism, especially understanding systemic diseases using systems biology approaches such as Flux Balance Analysis (FBA). The Recon approach incorporated the greatest number of biochemical reactions possible as well as transport processes to model human metabolism. Based on the Recon1 model, algorithms and applications for network reconstructions have been published subsequently [4]–[11].

Such algorithms have been applied to high-throughput, -omic data sets [12] defining tissue- or cell-specific metabolic networks and further elucidating genotype to phenotype relationship for single or multiple tissues [9], [10], [13], [14]. But understanding systemic metabolism requires not only individual tissues, but also their interlinked interactions [8], [15]. In complex organisms, different organs or tissues work synergistically to achieve overall metabolic homeostasis. This remarkable specialized division of labor among tissues is reflected in tissue-specific expression of genes coding for metabolic enzymes. On a whole-body level, the various tissues operate independently to some extent but at the same time also exchange metabolites through the circulation of body fluids thereby maintaining an overall metabolic homeostasis. Thus, metabolism of the whole organism has to be understood in terms of integrating tissue-specific metabolic pathways with interorgan metabolite transport. There have been several studies addressing this issue using FBA approach in which interorgan metabolic transports were inferred from the global analysis of tissue-specific metabolic networks in multiple tissues [8], [15]. In the present study, we take a different approach to determine metabolite transports based on the observation that transmembrane transporters and cognate enzymes form tightly linked local clusters, the so-called “membrane transport metabolon” [16]. In this view, the local couplings between transporters and their adjacent enzymes rather than the global metabolic networks play a pivotal role in metabolic transports. For this reason, we focus on solute carrier proteins (Slcs), which are transmembrane proteins transporting hundreds of polar metabolites required in many and diverse metabolic reactions. Slc transporters are tightly coupled to the enzymes that metabolize the transported solutes. Our interest is to seek evidence for Slc-mediated transport by its coupling with neighboring enzymatic reactions for each organ. We resorted to a computational approach to score local active metabolism/transport clusters based on gene expression, a strategy that is readily applicable to all organs. We used a well-defined scoring function that has already been applied in differential network analysis [17] to derive optimal local clusters. This method takes into account the metabolic connectivity and quantitative enzyme/transporter expression data. Using this method, we first identified and scored local metabolism/transport clusters for 17 major metabolically active organs in mouse based on gene expression profiling data of metabolic enzymes and metabolite transporting Slcs. We then estimated the connections between the 17 organs via predicted transports that would allow interactions between them through blood circulation. Finally, we demonstrated the usefulness of our approach to the understanding of whole-body metabolism by applying it to an important biological process: circadian rhythm.

## Results

### Identification of local metabolism/transport clusters

This study is concerned with local coupling between metabolism and the transport processes mediated by Slcs. The enzymatic reactions are derived from Kyoto Encyclopedia of Genes and Genomes (KEGG) database for mouse [18], which contains 1624 non-redundant reactions (Table S1) catalyzed by 1432 enzymes. KEGG pathway maps provide major metabolites that these enzymes act upon. Transport processes are derived from Slc Table found in the BioParadigms database (http://Slc.bioparadigms.org) containing the currently known Slcs and their transported metabolites [19]. After manual curation, 132 Slcs that are located on the plasma membrane are considered for interorgan transport. These Slcs transport a total of 136 metabolites listed in Table S2.

**Figure 1:**
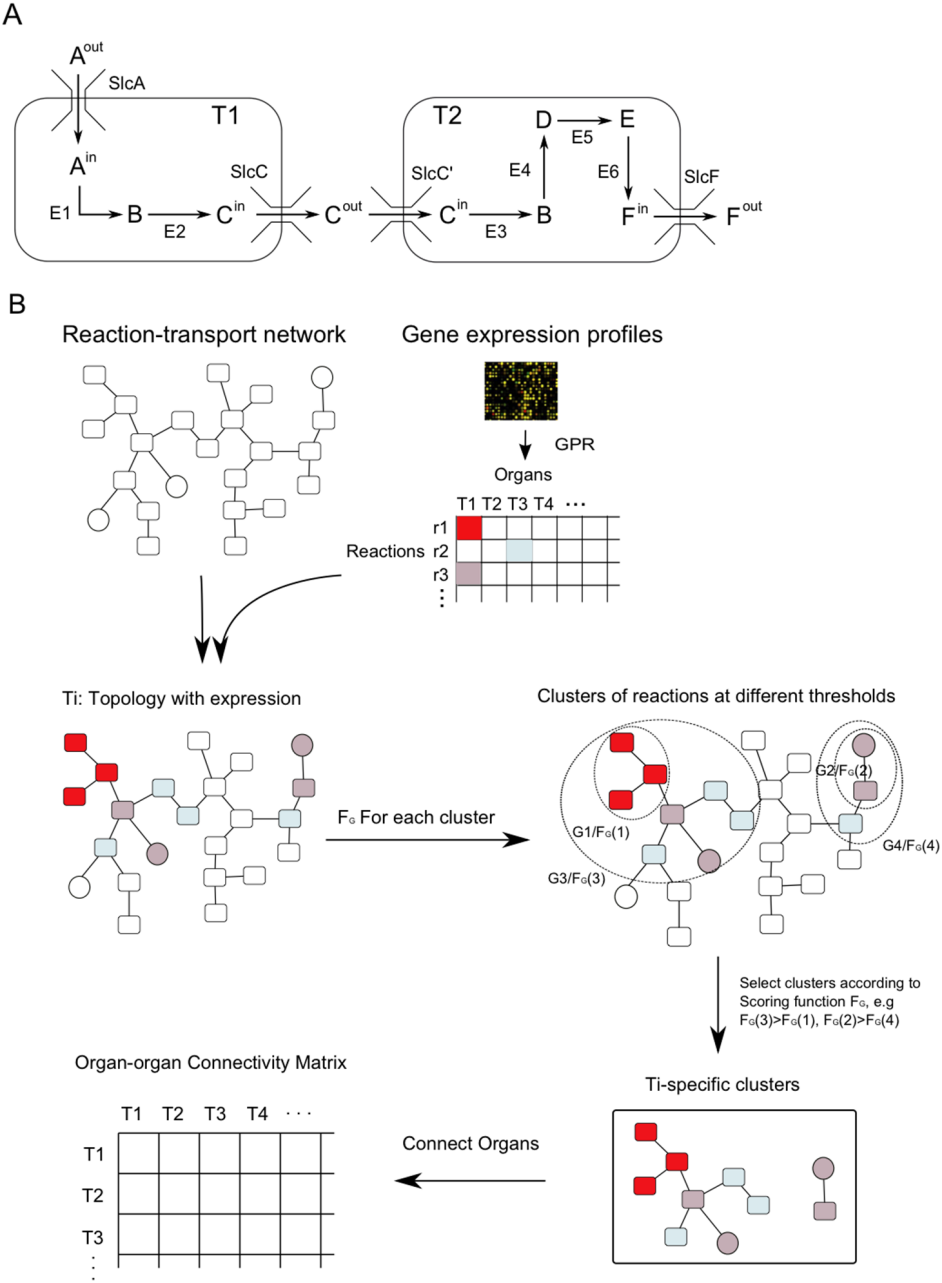
A model of a metabolism/transport network between organs and strategy to develop tissue-specific metabolism/transport clusters. (A) In each organ (T1 and T2) different metabolic processes may occur which are interconnected by interorgan metabolite transport mediated by Slcs. Alphabetic letters (A–F) represent metabolites catalytically converted by enzymes E1–E6. SlcA/C/C’/F are Slc transporters transporting metabolites A, C, F respectively. (B) Illustrates the procedure to derive tissue-specific metabolism/transport clusters. The reference network consists of enzymatic reactions (rectangular nodes), Slc-mediated transport reactions (circular nodes) and metabolites (lines connecting nodes). The score of a node is derived from the gene expression scores taken from microarray data and based on the Gene-Protein-Reaction (GPR) association. For a given organ Ti, node colors reflect high (red) to low (light blue) scores (Figure S1). Nodes connected by shared metabolites can form subclusters (dashed ellipses: G1, G2, G3, G4) based on a threshold for scores and each cluster obtains a score (F_G_(1) to F_G_(4)) according to the scoring function (F_G_). With progressively decreasing thresholds, increasingly larger groups of reactions and transport processes emerge (G1 becomes a part of G3 with lower threshold). After integrating all optimal subclusters (G3 and G2), tissue-specific clusters are obtained (lower right). Based on the derived tissue-specific clusters, organ-organ connectivity matrix is constructed (lower left).

Figure 1A makes the point that Slcs and metabolic enzymes can be linked through common substrates to carry out metabolic functions. Metabolic enzymes (enzymes E1 to E6 in Fig. 1A) and the cognate Slcs (e.g. SlcA is linked to E1 in Fig. 1A) can generate a local metabolism/transport cluster if Slcs and corresponding enzymes are both expressed. The overall strategy of our approach was to search for highly expressed clusters consisting of enzymatic and transport reactions. Figure 1B shows a scheme of our approach. Metabolic reactions (i.e. chemical conversion of compound A into compound B, Fig. 1A) are represented by rectangular nodes while transport processes (e.g. A^out^ to A^in^ in Fig. 1A) are represented by circles. The nodes are connected by edges whenever they share the same metabolite (Methods, Fig. 1B). The nodes and edges constitute the network in our study.

We derived quantitative expression scores of genes encoding the above mentioned components for a total of 17 metabolic organs in mouse (Table S3, see Methods). These scores reflect the extent to which a particular gene is specifically expressed in one of the 17 organs. Next, reaction scores were determined. If a single enzyme or Slc mediates the reaction or transport, then the reaction score was equal to the gene expression score. In the case of isozymes or when multiple Slcs could transport a particular solute, the reaction score was equal to the highest gene expression score (see Methods). This yields a network topology consisting of connected nodes, each with an assigned score. In Figure 1B (middle left scheme) the score levels are reflected by different shades from red to blue. The connected nodes in the network bearing scores above a given threshold form local clusters defined as components (e.g G1 to G4 in Fig. 1B middle right). By progressively decreasing the threshold, the clusters that were separated at a higher threshold will merge thereby forming even larger groups. The set of nested clusters under the series of decreasing thresholds is hierarchically ordered and forms a component tree, which can be computed in linear time [20]. The formation of this so-called component tree is illustrated in Figure S1. For each component G of the network topology (G is any one of the dashed ellipses in Fig. 1B middle right), we defined the scoring function 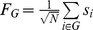, where 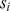 is the expression score for node 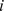 in G and N is the number of nodes in G. This scoring function takes into account the scores of individual nodes and the size of the cluster. It has also been commonly used in differential network analysis [21] to determine the optimal subnetwork from a whole network. Then we searched along each branch of the component tree from top-down for the optimal local metabolism/transport clusters in all branches that optimized the scoring function. As an example illustrated in (Fig. 1B middle right), there are two branches to be searched in the component tree. The left branch ends with G3 because the calculated score of G3 (F_G_(3)) is larger than the score of G1 (F_G_(1)). G2 becomes the optimal cluster with the higher F_G_ (F_G_(2)>F_G_(4)) in the other branches. Taken together, the collection of optimal local clusters from all branches of the component tree is considered as tissue-specific metabolism/transport clusters coupling enzymatic reactions with Slc-mediated transports (e.g Fig. 1B lower right). The objective is to obtain an organ-compound interaction matrix and, ultimately, an organ-organ connectivity matrix (lower left matrix in Fig. 1B) that can reflect the metabolic connections between organs.

### Evaluation of scoring function and local metabolism/transport clusters

We examined the effectiveness of the scoring function approach by computing the scores in each organ for 48 predefined reaction groups corresponding to well characterized metabolic reaction sequences (Table S4) using our scoring function. Table 1 lists the top 20 groups with the highest scores and the organs in which the groups would occur, based on their scores. Here the scoring function correctly assigned high scores for the reaction groups with known tissue-specific metabolic functions such as bile acid synthesis in liver and cortisol synthesis in adrenal gland.

**Table 1:** The 20 highest scoring predefined reaction groups.

Predefined reaction groups	Description	Organ	Score
Cholesterol = >Bile	Bile synthesis	Liver	18.3
Cholesterol = >Cortisol	Cortisol synthesis	Adrenal gland	16.2
Tryptophan = >Acetyl-CoA	Tryptophan degradation	Liver	13.9
Cholesterol = >Cortisol	Cortisol synthesis	Ovary	13.4
Tyrosine = >Acetoacetate + Fumarate	Tyrosine degradation	Liver	13.4
Tryptophan = >NAD+ (*de novo*)	de novo NAD+ synthesis	Liver	13.4
Lysine = >Acetyl-coA + Glutamate	Lysine degradation	Kidney	13.2
Lysine = >Acetyl-coA + Glutamate	Lysine degradation	Liver	11.8
Histidine = >Glutamate	Histidine degradation	Liver	11.4
Tyrosine = >Adrenaline	Adrenaline synthesis	Adrenal gland	10.8
Glucose = >Pyruvate	Glycolysis	Muscle	10.8
NH3+ Ornithine = >Citrulline	Citrulline synthesis	Liver	10.4
Tryptophan = >Acetyl-CoA	Tryptophan degradation	Kidney	10.2
Betaine = >Sarcosine	Sarcosine synthesis	Liver	10.0
Glucose = >Pyruvate	Glycolysis	Testis	10.0
Fatty Acids = >Acetyl-coA	Fatty acid oxidation	Liver	9.5
Tyrosine = >Noradrenaline	Noradrenaline synthesis	Adrenal gland	9.3
Citrate + Acetyl-CoA = >Citrate+2CO_2_	TCA cycle	Heart	9.2
Fatty Acids = >Acetyl-coA	Fatty acid oxidation	Kidney	9.1
Acetyl-coA = >Cholesterol	Cholesterol synthesis	Liver	9.0

Predefined reaction groups corresponding to metabolic reaction sequences were assessed by our scoring function in all 17 organs. The 20 highest scoring groups are shown in this table with their substrates and products listed in column 1 followed by a short functional description (column 2) and the identity of the tissue (column 3) with the highest score (column 4). For a comprehensive list of all predefined groups see Table S4.

**Figure 2:**
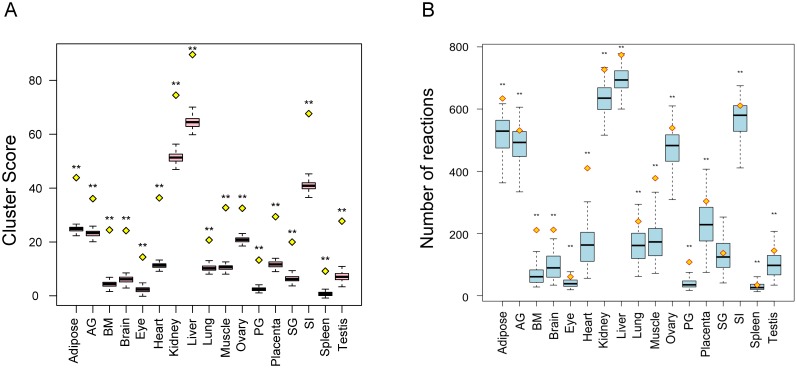
Evaluation of the statistical significance and the size of the largest connected local tissue-specific metabolism/transport clusters. (A) The yellow diamonds mark the scores of the largest connected clusters. The distribution of simulated scores of the same cluster is illustrated in the pink boxplot. (B) The yellow diamonds mark the sizes of the largest connected clusters. The sizes of the simulated largest connected clusters are illustrated in the form of a blue boxplot. AG, adrenal gland; BM, bone marrow; PG, pituitary gland; SG, salivary gland; SI, small intestine. ** represents p<0.001 in Student’s t-test.

We evaluated the statistical significances and sizes of the connected local metabolism/transport clusters in each organ. We calculated the statistical significances of our clusters by comparing their scores with the calculated scores from the same clusters of reactions with randomly permutated scores [22]. The statistical significances of all clusters with more than 20 reactions are listed in Table S5. For the largest clusters in 17 organs, their scores (yellow diamonds in Fig. 2A) are significantly higher than those calculated from the network nodes with randomly permutated reaction scores (p-value <2.2E-16, Fig. 2A). From the predicted local metabolism/transport clusters of organs, we observed that they fell into two types: small isolated clusters containing less than 10 reactions (enzymatic and transport) and large interconnected clusters containing more than 100 reactions. The sizes of the largest connected clusters, in some organs, such as adipose, kidney and liver, huge clusters (yellow diamonds in Fig. 2B) with as many as 600–700 reactions were identified. In other organs that do not have a prominent role in metabolism (eye, pituitary gland, and spleen), connected clusters are much smaller. We randomly permutated the scores of nodes for each organ for 100 times and computed the local metabolism/transport clusters for such simulated data. Although the numbers of nodes included in the optimal clusters from simulated data are similar to the real data clusters, the sizes of the largest connected simulated clusters were significantly smaller than the real data clusters in all organs except for salivary gland. These results indicate that our real data clusters are statistically significant and in general better connected than randomly permutated ones.

**Figure 3:**
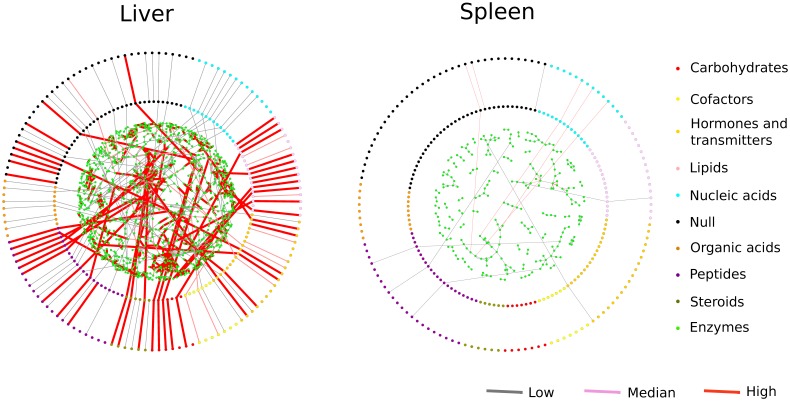
Tissue-specific metabolism/transport clusters in liver and spleen. Each organ possesses its own unique metabolism/transport clusters. The outer and inner circles line up all small molecule compounds that are transported by a Slc. Node color indicates compound types (see legend). The dots representing compounds are connected by a red line, if an appropriate Slc is expressed in the organ in question. The circular area in the center contains the reactions realized in each organ investigated. In these clusters, reactions that occur are marked with a red line. The above diagrams can be interactively viewed using Cytoscape (Data S1).

**Figure 4:**
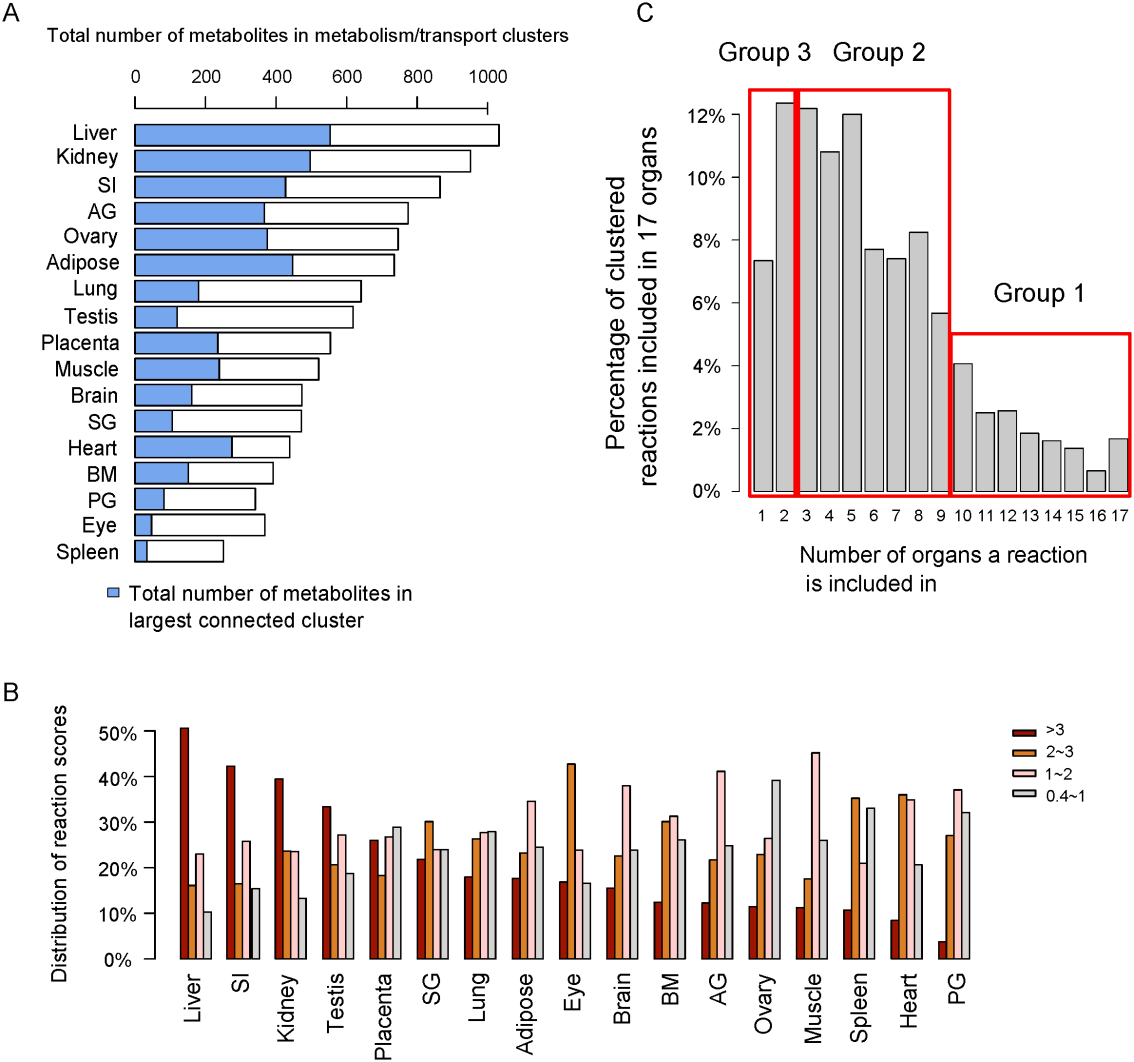
Comparing characteristics of tissue-specific clusters. (A) Shows the number of metabolites in the 17 tissue-specific clusters and the number of metabolites in the largest connected subclusters (blue portion of the bars). (B) The color-coded percentages of different magnitude of reaction scores in the tissue-specific networks for different organs. (C) The histogram indicates the percentage of reactions common to an increasing number of tissue-specific clusters. The x-axis is the numbers of organs that a reaction is included in tissue-specific clusters across 17 organs. Rectangles delineate groups of reactions that are tissue-specific (box marked as Group 3), reactions that are shared by a limited number of organs (box marked as Group 2) and ubiquitously occurring reactions (box marked as Group 1). AG, adrenal gland; BM, bone marrow; PG, pituitary gland; SG, salivary gland; SI, small intestine.

Figure 3 illustrates all local metabolism/transport clusters for liver and spleen that collectively represent substantial fractions of the whole body metabolism/transport. The overview of 17 organs is included in Figure S2. These metabolism/transport clusters can be explored interactively using the Cytoscape software (see Data S1). In this scheme, the dots on the outer and inner circles represent the 136 Slc-transportable compounds that are sorted and color-coded according to their chemical characteristics (nucleosides, lipids etc.). The compounds located on the outer circle are only connected to their inner ring counterpart if a Slc is expressed that can transport the compound in question. The innermost area represents all enzymatic reactions that can convert metabolites within a given organ. The scopes of transports and enzymatic reactions significantly vary between the organs (see Fig. 3). For example, liver, kidney and small intestine contain the highest numbers of metabolites and are home of the largest clusters of connected metabolites (Fig. 4A). Figure 4B illustrates that the reaction score distributions vary significantly between organs for these selected local metabolism/transport reactions. In particular, kidney, small intestine, and liver possess 40% to 50% highly expressed reactions with a score >3 (i.e. enzyme and/or Slc transporter mediating a process is strongly expressed) that form local metabolism/transport clusters. By contrast other organs, such as ovary, possess only 10% to 20% of highly expressed reactions within their reaction clusters. Taken together, local metabolism/transport clusters in different organs showed significant differences not only in size but also with regard to the expression levels of their constituents, the metabolic enzymes and Slcs. A histogram (Fig. 4C) illustrates how often different organs share the same reactions within their clusters. Essentially, the number of reactions widely shared among organs is very limited. Less than 2% of the reactions are common to all 17 organs.

We subdivided the histogram into three groups based on organ specificity (red boxes in Fig. 4C) and asked which reactions typically occur in each group. Group 1 contains 205 reactions that appear in at least 10 organs. These reactions (Figure S3A) cover purine metabolism (25 reactions), pyrimidine metabolism (13 reactions), glutathione metabolism (11 reactions), glycolysis/gluconeogenesis (9 reactions), glycerolipid metabolism (9 reactions), and the pentose phosphate pathway (6 reactions). Reactions shared among 3–9 organs (group 2) are tabulated in Figure S3B. They are involved in fatty acid metabolism, fatty acid elongation, fatty acid biosynthesis, and the TCA cycle co-expressed by liver, kidney as well as heart and muscle. These reactions/pathways generally have central roles in metabolism, such as providing starting materials for protein biosynthesis and energy generation. Group 3 contains reactions occurring in only one or two organs and represents highly tissue-specific functions such as steroid hormone biosynthesis (35 reactions), bile acid biosynthesis (20 reactions), steroid biosynthesis (16 reactions), tryptophan metabolism (11 reactions), tyrosine metabolism (7 reactions), and glycine-serine metabolism (6 reactions). Figure S3C shows several examples of such reactions.

### Classification of Slc-mediated transports

**Figure 5:**
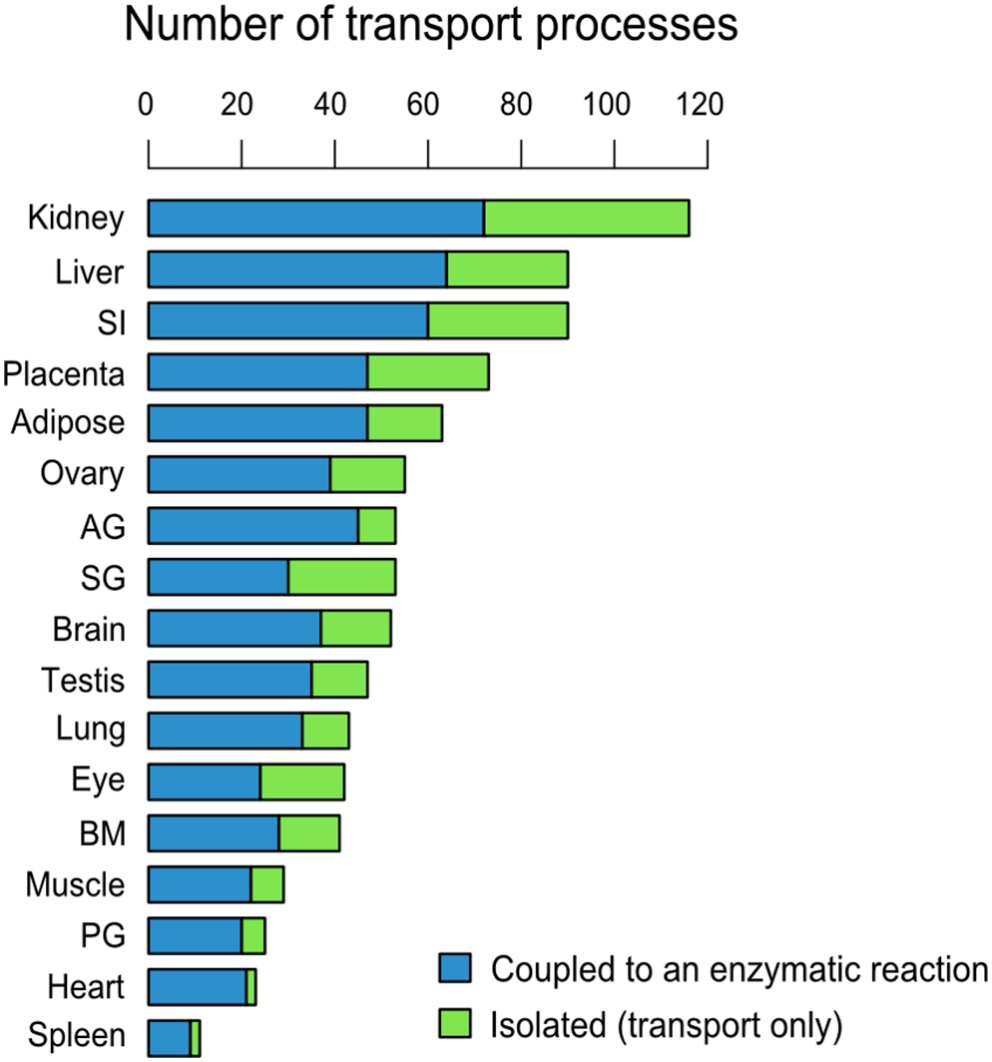
Distribution across 17 organs of the two classes of Slc-mediated transport modalities. The blue bars represent the number of enzyme-coupled Slc transport processes while the green bars represent the number of Slc-mediated transport processes that are not linked to an enzymatic reaction. AG, adrenal gland; BM, bone marrow; PG, pituitary gland; SG, salivary gland; SI, small intestine.

We next examined Slc-mediated transport processes that connect the two outer circles of Figure 3 and Figure S2. These processes play an important role in interlinking organs. We observed that in many instances transported solutes can be directly linked to an enzymatic reaction represented in the innermost area that form local metabolism/transport cluster. In other instances, such could not be observed. Therefore, we divided Slc-mediated transport into two classes. Class 1 includes cases where transported solutes are directly coupled to at least one enzymatic reaction requiring that Slc and coupled enzyme are locally expressed in the same organ. These coupled transports are responsible for metabolic absorption for downstream metabolism or secretion from upstream synthesis. In contrast, class 2 includes predicted cases where the transport process is isolated. These cases are responsible for directly solute transport, such as excretion and reabsorption of metabolites in kidney. This class can also be due to either the gaps between transports and enzymatic reactions in our network or the coupling with enzymatic reactions of very low expression in the organ of interest. For all analyzed organs the distribution of these two classes of Slc-mediated transport is shown in Figure 5. Transport processes are highly active in kidney, liver and small intestine. The isolated transport reactions in class 2 are enriched in epithelia tissues such as kidney and small intestine (see Fig. 5 green parts). This indicates that our algorithm is effective in identifying isolated transports from metabolism. Examples of class 1 and class 2 transports with top expression scores are provided in Table 2. The table states the class of transport, the name of the transported solutes, the organ and Slc with the highest expression score for the transport, and in the case of class 1 also the enzymes associated with one or several of the transported solutes.

**Table 2:** Top scoring Slc-mediated transport processes by class

Class	Transported solutes	Organ	Slc	Score for Slc	Coupled enzymes
1	Arachidonic acid, Linoleic acid, Myristic acid, Palmitic acid	Liver	Slc27a5	8.48	Acsl1, Cyp2c29, Cyp3a11, Fasn
1	Arachidonic acid, Linoleic acid, Palmitic acid	Kidney	Slc27a2	8.31	Acot3, Cyp2j5
1	Glucose, Galactose	Small Intestine	Slc5a1	7.99	Lct, Mgam
1	Aspartate, Glutamate	Kidney	Slc7a13	7.92	Acy3, Ggt1
1	Cholic acid, Glycocholate, Taurocholate	Liver	Slc10a1	7.56	Baat
1	Alanine, Arginine, Cysteine, Glutamine, Glycine, Histidine, Lysine, Methionine, Serine	Liver	Slc38a4	7.24	Aass, Agxt, Arg1, Cth, Gls2, Hal, Mat1a
2	Mannose	Liver	Slc2a2	7.18	
2	Estrone 3-sulfate	Liver	Slco1b2	7.07	
2	Alanine, Methionine, Asparagine	Placenta	Slc38a4	7.04	
2	Urate	Kidney	Slc22a12	6.92	
2	Lysine, Cystine	Small Intestine	Slc7a9	6.91	
2	Glucosamine, Mannose	Kidney	Slc2a2	6.75	

Class 1 and class 2 are Slc-mediatesd transport processes with and without enzyme coupling, respectively. The six highest scoring transport processes of each class are shown. The directly coupled enzymes for class 1 transpsort are listed in column 6. See also Table S6 for more examples.

An example for class 1 transport is that of fatty acids mediated by Slc27a5 in liver and Slc27a2 in kidney. Both these organs have high expression scores for downstream enzymes involved in fatty acid metabolism. Amino acids such as histidine and alanine are transported by Slc38a4 in liver where the appropriate metabolizing enzymes are also expressed (Table 2). Additional examples for class 1 can be found in Table S6. To name just a few, there is (1) the enzyme-coupled Slc transport of phenylalanine and tyrosine in liver and kidney, where phenylalanine is taken up by Slc16a10, Slc6a19, or Slc7a8 and converted by Pah to tyrosine which is then exported by Slc16a10 or Slc7a8. (2) The transport and metabolism of branched-chain amino acids which enter the brain via Slc6a15 and serve as a substrate for Bcat1. (3) The uptake of lysine in liver by Slc7a2 or Slc38a4 followed by liver-specific degradation.

Class 2 transport is the type of transport processes that merely transport solutes across an epithelial boundary (Fig. 5). For example, transports belonging to class 2 are enriched in kidney, which reflects the physiological function of kidney that is to excrete waste products and to reabsorb essential nutrients such as glucose and amino acids [23]–[26]. We found from our kidney metabolism/transport clusters that asparagine, myristic and arachidic acid as well as vitamins were reabsorbed by kidney. We also noted that in small intestine there are transport processes for branched-chain amino acids such as isoleucine and valine by Slc6a19 and lysine and cysteine by Slc38a4 apparently without enzyme coupling.

A comprehensive list of Slcs, their transportable compounds and whether they are engaged in class 1 or class 2 transports is found in Table S6.

### Estimation for interorgan metabolic connectivity

**Figure 6:**
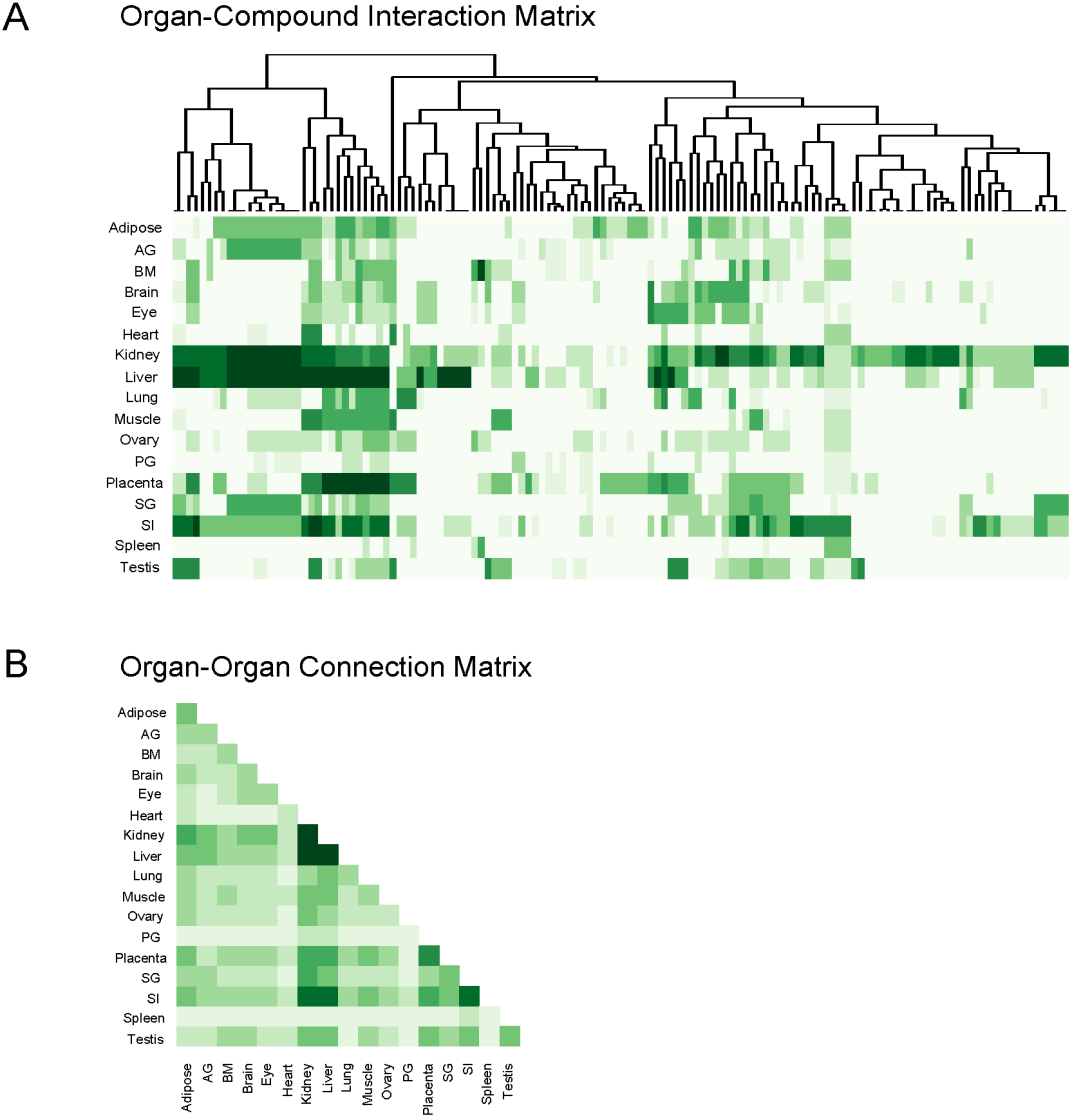
Organ-Compound interaction matrix and Organ-Organ connectivity. (A) Rows are 17 organs. Columns are 136 transport processes. Cell colors reflect high (dark green) to low (white) transport capabilities of corresponding metabolites for a given organ. (B) Cell colors reflect high (dark green) to low (white) connectivity via Slc-mediated transports between organs. AG, adrenal gland; BM, bone marrow; PG, pituitary gland; SG, salivary gland; SI, small intestine.

Based on the local metabolism/transport clusters for 17 organs consisting of transport processes and enzymatic reactions, we tried to infer the metabolic interactions between organs. We considered the 136 Slc-transportable metabolites as possible linkage points between organs. We first identified the local metabolism/transport clusters involving the transport processes of these metabolites and used their corresponding optimal scores of the clusters deriving from scoring function as the measure of the transport capability of the metabolites for the organs. The score is zero if the transport process of the metabolite cannot be found in the local metabolism/transport cluster of this organ. Thus we obtained a 17 by 136 matrix (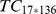) to estimate the organ transport capability for all transportable metabolites (Fig. 6A, Figure S4). It can be seen that few metabolites can be transported in spleen but transports are highly active in liver and kidney. The organ-organ connectivity matrix 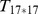 can be obtained by computing the product of 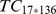 with its transposed matrix. Therefore, this connectivity matrix between organs has taken into account all transports mediated by Slcs and their coupled enzymatic reactions. Figure 6B shows the connectivity matrix between organs in the form of heatmap. The diagonal element of the matrix represents the overall metabolism/transport activity of all Slc-transportable metabolites for a given organ. We found that kidney, liver, and small intestine have the highest scores while spleen has a very low score. In terms of interorgan metabolite transports, we found that kidney and liver as well as small intestine are closely interlinked with each other through metabolic transports. In addition, placenta is also highly connected with other organs potentially representing the exchange of metabolites between fetus and mother. But much lower exchanges were found between organs such as spleen or heart. We further evaluated the organ connectivity via different types of transportable metabolites by dividing the metabolites in 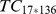 into sub-matrices for metabolites of distinct types. The organ-organ connectivity matrices for different types of metabolites are calculated accordingly and shown in Figure S5. We found that there are significant differences in interorgan metabolite transports between different types of metabolites. For hormones including neurotransmitters, testis and brain, the main producing organs, are connected with kidney and liver that are main organs for excretion and detoxification of hormones (Figure S5B). For lipid metabolites, the main connection was found between adipose tissue, the organ for lipid deposit, and liver, the organ for lipid catabolism (Figure S5C). Taken together, the connectivity matrices in Figure 6 and Figure S5 provide us direct and quantitative estimates of potential metabolic interactions between organs.

From the predicted interorgan interactions, we found that some have been previously known in the literature. For example, the well known cori cycle [27] between skeletal muscle and liver can be included by our method. Muscles absorb glucose from blood and produce lactate that in turn is taken up by liver in order to regenerate glucose through gluconeogenesis. The similarly organized glucose-alanine cycle exchanges glucose and alanine between liver and muscle (Figure S6A). The urea cycle [23], [28], [29] between liver, kidney and small intestine can also be recapitulated in our organ interactions (Figure S6B). The synthesis of tyrosine from phenylalanine, which has been observed in liver and kidney [30]–[33], also occurs in our predictions. We further observed that the product of tyrosine degradation can be used for TCA cycle and fatty acid synthesis in liver and kidney, consistent with previous observations [34]. In addition to liver and kidney, enzyme-coupled Slcs for tyrosine and phenylalanine were also expressed in adrenal gland and muscle. In adrenal gland, the transport of tyrosine can feed into the synthesis of adrenaline from tyrosine. There are evidences for the release and uptake of branched-chain amino acids in kidney under different conditions [23], [28], [32]. From our prediction, their degradation with active transports was specific in kidney, adipose and adrenal gland. Small intestine only showed direct active transports of branched-chain amino acids likely through absorption from food intake. But brain and placenta can take up and metabolize the branched-chain amino acids with enzyme-coupled Slc transport. These observations are consistent with the current knowledge that branched-chain amino acids are only metabolized in extra-hepatic tissues. From our prediction, we found that serine de novo synthesis by Phgdh, Psat, and Psph was only expressed in adipose but absent in liver and kidney. In contrast, enzymes involved in serine glycine conversion, glycine degradation by glycine cleavage enzyme, and glutathione synthesis from glycine were exclusively expressed in liver and kidney. Therefore, we predict that serine may be involved in an inter-organ transport in which adipose synthesizes and releases serine as the precursor of glycine for liver and kidney [32], [35], [36] (Figure S6C). Evidently, this novel pathway is still hypothetical and needs to be experimentally validated using isotope labeled compounds.

We examined the measured concentrations of Slc transportable metabolites in human blood derived from Human Metabolic Database (HMDB) [37]. As shown in Table S7, most of the Slc transportable metabolites that we predicted for interorgan metabolic transports were found in human blood. This supports the notion that these metabolites can be transported between organs through blood circulation. We compared our predicted transports with the ones predicted from Bordbar’s work [15] between liver, adipose and muscle based on global reconstruction of metabolic networks. We found that 82.6%, 72.7% and 57.1% of Slc transports occurred in Bordbar’s results can be recaptured by our method in adipose, liver and muscle, respectively. In addition, we found that a large amount of transports predicted from our method are absent from Bordbar’s study (Figure S7A). Many of these unique transports in our result have support from literature and previous experiments (Figure S6). For example, hormones and neurotransmitters such as serotonin and cortisol are known to be actively transported into liver for deactivation [38]. These transports can be identified from our result but absent from Bordbar’s result. The transports in these three organs exclusively predicted by our method are provided in Table S8. We also compared our result with Shlomi et al’s result [8], which derived a global map of secretion and uptake of 249 metabolites across different tissues when they reconstructed tissue-specific metabolic networks. 46% of their 249 transportable metabolites are assigned with known membrane transporters. 88 out of 115 metabolites are transported by Slcs. Among these 88 metabolites, 75 of them have been annotated by our compound-SLC relationship (Figure S7B). Shlomi et al. predicted 81 and 71 transport processes for kidney and liver respectively from these 249 metabolites. We inferred 116 and 90 transport processes for kidney and liver respectively, which showed significant overlaps with Shlomi’s result (Fisher’s exact test, p-value = 6.44e-08 for kidney, p-value = 1.09e-05 for liver) from our 136 transportable metabolites. In short, our simple method can not only recapture previously known Slc-mediated transports, but also predict new metabolic transports compared to the results from global reconstruction of metabolic network.

### Application in circadian metabolism

Currently, many metabolomic studies have been conducted in body fluids such as blood. Our method has the potential to link the changes of metabolites in the blood with the changes of gene expression in the tissues that are exchanging metabolites with the blood. We chose a well-studied physiological process, circadian rhythm, to test the applicability of our method. It has been known that circadian clock plays a key role to control our daily metabolism [39]. Circadian transcriptomic studies have provided genome-wide circadian gene expression in various organs [40]. Minami et al.’s metabolomic study has found metabolites whose levels showed circadian oscillation in the blood [41]. In the circadian gene expression data collected from our own meta-analysis study [40], we systematically searched for the genes showing circadian gene expression among our predicted metabolism/transport clusters in liver, kidney, and adipose respectively. Among the 26 identifiable metabolites that were found to oscillate in blood in Minami et al’s data, 13 of them are transportable metabolites included in our study. We have found circadian oscillating enzymes or Slc transporters in our clusters linking to the circadian oscillations of glutamine, proline, leucine, glycine and methionine levels in the blood (Table 3). In our adipose and kidney clusters, Glul synthesizing glutamine from glutamate showed circadian oscillation with the peak at circadian time CT15 (circadian time 15). Glutamine is known to be synthesized in extra-hepatic tissues. This result is consistent with the circadian peak of glutamine level at CT18 in the blood. In our kidney cluster, Slc7a7 peaking at CT6 can transport leucine for its incorporation into protein by leucine-tRNA synthetase, Lars, also peaking at CT7. In our adipose cluster, Bcat1 peaking at CT4 is a key enzyme for leucine degradation. The circadian uptake of leucine in kidney and adipose may lead to the depletion of leucine at CT7 and the peak at CT19 in the blood. In our liver cluster, the circadian uptake of glycine mediated by Slc6a9 peaking at CT13 coincides with the peak of glycine level at CT12 in the blood. This may supply glycine for its later degradation catalyzed by Gldc peaking at CT23. In addition, we have found that one of our liver clusters is significantly enriched with circadian oscillating genes (Fisher’s exact test, p = 0.006). In this cluster, two Slc transporters, Slc22a1 peaking at CT6 for estradiol and Slco1b2 peaking at CT9 for estradiol-17beta 3-glucuronide, together with all enzymes except one are circadian oscillating in liver. This liver cluster is responsible for the degradation of estrogen-related steroid hormones. It is known that the level of steroid hormone such as cortisol is circadian oscillating [42]. Previous study only focused on their circadian synthesis. Our result suggests that the circadian absorption and degradation of hormones in liver may also be important for their circadian turnover in the blood. In summary, by applying our method to another dataset of circadian rhythm, we show that our method is valuable to obtain new biological insight of the inter-organ connectivity in metabolism.

**Table 3:** Circadian transportable metabolites in the blood with the corresponding circadian enzymes or transporters

Metabolites in the blood	Reaction/Transport	Adipose	Kidney	Liver
Glutamine (17.7)	Glutamate->Glutamine	Glul(16.8)	Glul(15.0)	
	Glutamine->Carbamoyl-P	Cad(10.3)		
	Glutamine transport		Slc7a7(6.7)	
Leucine (18.9)	Leucine->4-Methyl-2-oxopentanoate	Bcat1(3.7)		
	Leucine->L-Leucyl-tRNA		Lars(5.7)	
	Leucine transport		Slc7a7(6.7)	
Glycine (12.1)	Glycine->CO2+S-Aminomethyldihydrolipoylprotein		Gldc(15.8)	Gldc(23)
	Glycine <-> Serine		Shmt1(1.7)	
	Glycine transport			Slc6a9(13.5)
Proline (18.3)	Peptide->Proline		Lap3(4.8)	Lap3(23.2)
	Proline->4-Hydroxy-L-Proline	P4ha1(18.4)	P4ha1(18.7)	
Methionine (18.8)	L-Homocysteine->Methionine			Bhmt(2.2)
	Methionine->4-Methylthio-2-oxobutanoate			Tat(17.1)

The numeric numbers in the parentheses are circadian peak times in CT (circadian time) of circadian metabolites in the blood or circadian oscillating genes in adipose, kidney, and liver.

## Discussion

In this study, we estimated the metabolic connectivity between 17 major metabolic mouse organs using a simple computational approach. The prediction is based on the local coupling between metabolic enzymes and Slc transporters obtained from a combination of quantitative expression data and the KEGG metabolism/transport network. A computational method to derive local metabolism/transport cluster was implemented by optimizing a scoring function along a data structure called component tree. This was constructed by gradual lowering the threshold for the expression scores of sub-networks. We calculated a score for each constructed sub-network. We were able to obtain optimal local metabolism/transport clusters linking metabolic reactions or Slc-mediated transports according to the calculated scores for each of the 17 organs. In these clusters, we found that metabolic reactions were frequently tied to Slc-transporters that catalyze metabolite uptake and release. Other Slcs, however, mediate metabolite transport across epithelia without any association to metabolic enzymes. The current knowledge about Slc transport specificity is mostly obtained from *in vitro* studies in which a Slc is overexpressed and solute affinity and rate of transport are measured. These studies reveal a certain degree of promiscuity of Slcs. Particular Slcs can transport several structurally related solutes and, moreover, particular solutes can be transported by several Slcs. The local metabolism/transport cluster associates Slcs with enzymes of known substrate specificity. Such an association helps in delineating the physiological solutes that Slcs will actually transport in a given organ environment. Highly expressed Slc transporters can be viewed as entry and exit gates controlling metabolite exchange between organs and the body fluids. The local coupling that we identified between Slcs and metabolic enzymes is in line with the concept of membrane transport metabolons proposed by Moraes *et al.* [16], in which transporters and enzymes that metabolize the transported compounds may be physically associated. Such linkage in combination with the law of mass action may give rise to directionality of metabolic flux and facilitate more efficient and selective transport *in vivo*.

Our local metabolism/transport clusters linking metabolism to transport allowed the identification of previously uncharacterized exchange of metabolites between organs. Physiologists have long recognized the importance of interorgan metabolite transport [27], [43]. From the perspective of systems biology, the brain metabolism reconstruction [44] and the host-pathogen interactions of *M. tuberculosis* [45] have highlighted the importance of analyzing intercellular interactions [13]. However, most of these systems biology studies reconstructed metabolic network with data taken from literature and enhanced by manual modification. The FBA approach for reconstruction generally encounters 15% to 25% of highly expressed reactions to be isolated in each organ when using the Recon 1 network. The missing information made the interorgan network construction a daunting task. Here we proposed a local approach to seek evidences for active transports from highly expressed Slcs and adjacent metabolic enzymes. This approach circumvents the global reconstruction of tissue-specific metabolic networks. In this regard, KEGG is already sufficient to provide highly reliable information of major enzyme substrates to be paired with Slcs. Based on the literatures and Slc Table, we manually curated the relationship between Slcs and compounds. This makes the Slc-compound relationship in our study more accurate as compared to Shlomi et al’s study in which Slc-compound relationship was obtained without further curation from the global network in BiGG database [8]. In the present approach, we can identify transports with scores that coupled with only a few downstream or upstream enzymatic reactions or even isolate transports. Such transports are often neglected or considered as gaps because they are unable to generate fluxes in FBA used for the global reconstruction of metabolic networks. Slc-mediated transports provide an opportunity to extend our knowledge of interorgan metabolite transport that has been proposed years ago [43]. Furthermore, we used the scores of local metabolism/transport clusters to systematically estimate the metabolic interactions between organs (Fig. 6). From the organ-compound interaction matrix, we can identify the well-known cases where a compound synthesized in one organ and released by this organ is then taken up by another organ via a Slc and then funneled into enzymatic reactions within the second organ. Besides some examples mentioned above, we also made many interesting observations from our predicted clusters. For instance, one of glucose transporters, Slc2a1, expressed in placenta is probably responsible to bring in glucose to fetus. While the other glucose transporter Slc2a3 expressed in brain, may function for glucose uptake for neurons. We observed that fatty acid synthesis genes are highly expressed in adipose whereas the genes for fatty acid oxidation are expressed elsewhere such as liver, kidney and muscle. The absorption and excretion of fatty acids are predicted to be carried out by various Slc27a family transporters from their expression. Conversion of testosterone to estradiol-17beta is facilitated in ovary and testis. We can observe the significant expression of transporter (Slc22a3) of estradiol-17beta in ovary. Meanwhile, small intestine can convert estradiol-17beta to estriol with the estradiol-17beta uptake mediated by Slc22a1 from our prediction. This is potentially another inter-organ transport between ovary and small intestine. Such transports of estradiol-17beta may play an important role in the inactivation of the hormones. Similar transports may also exist for neurotransmitters such as serotonin synthesized in the brain then transported to liver for inactivation.

In this study, we focused on Slc-mediated transport because this transporter superfamily supplies cells with many of the basic building blocks required by cell metabolism. Nevertheless, some small molecules, critical for metabolism, use other means of transport. For example, free diffusion is the major mechanism of transport for gases, such as oxygen, and hydrophobic compounds cross membranes by diffusion. Endocytosis and exocytosis are other means of transport and translocate macromolecules such as proteins, hormones (insulin) and signaling molecules (dopamine) in and out of cells. ABC transporters are also a family of membrane bound transporters that translocate substrates such as lipids, sterols and drugs. In the present approach, these additional mechanisms of transport are not included but can be eventually incorporated using the same methodology developed for our study. Most importantly, our study tried to reveal an extensive system of interorgan metabolic transport and has shed light on the molecular players therein. In the end, metabolism has to be understood not only at the level of single organs but as an orchestrated process involving the entire body. In the case of single cell organism this issue does only arise at the level of intraorganelle transport; much remains to be done to address interorgan and intertissue transport in multicellular and multiorgan systems. Our work provides a novel attempt to bring together physiology and cellular biochemistry to tackle this important problem.

## Methods

### Definition of reference network

We downloaded all enzymatic reactions and their associated genes, i.e. Gene- Protein-Reaction (GPR) association, for mouse included in the KEGG database. We obtained the major metabolites participating in these reactions and the direction of reactions by parsing the KEGG pathway maps. By literature curation, we classified the Slcs by their subcellular localizations into plasma membrane, mitochondrial membrane, vesicular membrane, and other organelle membrane transporters. Only Slcs located on plasma membrane are considered in this study. Slc-mediated transport reactions were considered as reversible reactions. We then integrated all enzymatic reactions and Slc-mediated transport reactions into one reference network. In our reference network of metabolite transport, the nodes represent reactions including both enzymatic reactions and Slc-mediated transport reactions.

### Definition of scores for metabolism/transport reactions

To obtain reliable expression scores for metabolic enzymes and Slc transporters across mouse organs, we integrated three adult mouse organ microarray datasets from Gene Expression Omnibus (GEO) database: GSE10246 (BIOGPS mouse4302 arrays), GSE1133 (BIOGPS gnf arrays), and GSE9954 (mouse4302 arrays). We selected 17 organs that are common to all three datasets: adipose, adrenal gland, bone marrow, brain, eye, heart, kidney, liver, lung, musscle, ovary, pituitary gland, placenta, salivary gland, small intestine, spleen, and testis. We averaged the data from various brain regions in the first two datasets as the expression data of the whole brain. Among the selected organs, original expression values were first log10-transformed and then subtracted from the median for each gene as the scores of organ-specificity in every dataset, respectively. The total score of a gene in a given organ is the sum of scores from three datasets (Table S3). The score of a reaction, i.e. the node in reference network, is the maximum score of the genes associated with the reaction. In order to calculate the significance of predicted tissue-specific metabolism/transport cluster, we randomly re-assign reaction scores within an organ for 100 times to calculate the p-value.

### Predefined metabolism/transport clusters for validation

To obtain predefined metabolism/transport clusters, we manually curated KEGG modules corresponding to known anabolic or catabolic functions. They were selected for well characterized metabolic reaction sequences involving Slc transportable compounds as either substrate or product. The redundancy in KEGG modules caused by overlaps of reactions was removed. More predefined metabolism/transport clusters containing at least two consecutive reactions were added from literatures and their reaction sequences were identified in KEGG. In total, we obtained 48 predefined metabolism/transport clusters as shown in Table S4. We represented each predefined cluster by a summarized overall reaction containing substrate and product.

### The pseudo-code of our algorithm

A pseudo-code for the algorithm is described as follows:

The whole reference network can be represented by GN = (*M, Re*), in which *M* is a set of edges (metabolites) and *Re* is a set of nodes (reactions). 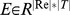 is a matrix denoting the node scores in *T* organs. Starting at a high score with threshold 3, we decrease the threshold gradually with a step size 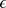 = 0.1. Each step corresponds to a set of connected clusters (e.g in Fig. 1B middle right, Set1 = (G1), Set2 = (G2), Set3 = (G3, G4)). For organ t:

For n = 0 to 26

threshold(n) = starting threshold –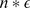 (threshold from 3 to 0.4)

Groups(n) = ReactionGroup(GN, E(t), threshold(n))

The procedure *ReactionGroup* computes the sets of connected subnetworks, defined as components, with node scores above threshold(n).

For n = 1 to 26

Branch_Step(n) = traceback(Groups(n))

Return ComponentTree

The procedure *traceback* is to trace the components under previous threshold for Groups(n). After tracing step by step, the hierarchical relationship of components forms a data structure called component tree (Figure S1). This structure simplifies metabolic network topology, taking a group of consistently highly expressed reactions as one component. It is aimed to reflect how closely connected between nodes according to network topology and expression.

For each branch 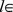 ComponentTree

set 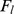 = 0

For each component 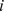 from top to bottom
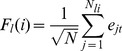


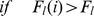


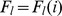




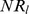
 = Nodes belonging to component *i*


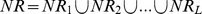
 (L is the number of branches).

Here 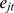 is the score for node 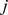 in organ 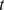 and 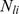 is the number of nodes in the component 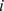 of branch 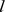. We searched for the component of branch 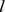 that possesses the largest F score in that branch and then integrated the optimal components derived from all branches to form the final set of tissue-specific network NR.

### Organ-compound interaction matrix and organ-organ connectivity matrix

We collected the Slc-mediated transport processes for each organ according to the derived local metabolism/transport clusters. The columns of organ-compound interaction matrix are the 136 Slc-transportable metabolites. The value of each cell in this matrix is the derived score of corresponding local metabolism/transport clusters in that organ. Then we calculated the product of the organ-compound interaction matrix and its transpose to represent the organ-organ connectivity. This connectivity matrix shown in Figure 6 considered all possible transportable metabolites between two organs while connectivity matrix for a given type of metabolites were derived similarly by restricting the columns in organ-compound interaction matrix to only the metabolites in that type.

## Supporting Information

Figure S1 **A schematic representation of the component tree.** A component tree is a data structure representing the hierarchical relationship between components (rectangular boxes) obtained with the decreasing score threshold (see main Text). Components are connected nodes in the reference network with scores above a given threshold. The top of the component tree consists of the nodes with highest scores. At each step of decreasing threshold, new nodes are incorporated into the components and new components are generated. The numbers indicated on the components represent the numbers of nodes within the components. The numbers next to the arrows represent the numbers of new nodes added into the component as the threshold decreases. The colors of components ranging from red to light blue represent the decreasing threshold values of the scores.
(PNG)

Figure S2 **Complete overview of Tissue-specific metabolism/transport clusters.** This is a complete collection of tissue-specific metabolism/transport clusters of all 17 organs investigated in our study. The detailed information of these diagrams can be found in the legend of Figure 3.
(PDF)

Figure S3 **Three different types of expression patterns of reactions in metabolic pathways across organs.** The metabolic reactions that are ubiquitous in at least 10 organs (A), restricted to 3–9 organs (B), or highly restricted to only one or two organs (C). The score of each reaction in the 17 organs is provided. The reactions are marked in red color for organs with high scores. The reactions in (A) and (B) belonging to different metabolic pathways form clusters (rectangular boxes with different colors) such as purine metabolism, pyrimidine metabolism and so on. (C) Heme biosynthesis pathway mainly occurs in bone marrow and hormones such as estrone can be synthesized by the ovary and adrenaline synthesis is restricted to the adrenal gland. The synthesis of niacin (vitamin B3) from tryptophan is restricted to liver. Calcidiol biosynthesis from vitamin D3 is specific for liver while its conversions into calcitriol and secalciferol are specific for kidney.
(PDF)

Figure S4 **Organ transport capability for all transportable metabolites.** Rows are 17 organs. Columns are 136 transport processes. Cell colors reflect high (dark green) to low (white) transport capabilities of corresponding metabolites for a given organ.
(PDF)

Figure S5 **Organ-organ connections via specific transportable metabolites.** 136 Slc-mediated metabolites are classified into seven categories: (A) Carbohydrates; (B) Hormones and transmitters; (C) Lipids; (D) Nucleic acids; (E) Organic acids; (F) Amino acids; (G) Steroid. Each heatmap matrix reflects organ-organ connection through a certain type of transportable metabolites. Cell colors reflect the degree of connectivity between organs.
(PDF)

Figure S6 **Selected examples of interorgan metabolism/transport processes and predicted transporters.** (A) Cori Cycle and Glucose-Alanine Cycle between liver and muscle. (B) Urea Cycle is realized in full in liver but only partially in kidney and in small intestine. This leads to the transport of citrulline and arginine between small intestine and kidney in the so-called citrulline-arginine shunt. (C) Serine synthesis in adipose and serine-glycine conversion in liver and kidneys may lead to the transport of serine between adipose and liver and kidney. Gln, glutamine; ORN, ornithine; CP, carbamoyl phosphate; CIT, citrulline; ARG, arginine; AS, aspartate; 3-PG, 3-Phosphoglycerate; 3-P-OH-Pyr, 3-Phosphonooxypyruvate; O-P-L-Ser, 3-Phosphoserine; Gly, Glycine.
(TIF)

Figure S7 **Comparison of predicted Slc-mediated transports.** (A)Comparison of predicted Slc-mediated transports between Bordbar’s result [15] and ours in adipose, liver and muscle. (B) Comparison of predicted Slc-mediated transports between Shlomi et al’s result and ours in liver and kidney [8].
(PDF)

Table S1 **Enzymatic reactions.** Column 1 is the reaction indexes assigned by us. Column 2 indicates the path numbers that reaction belongs to. Column 3 shows the reaction direction. Column 4 is the reaction ID assigned by KEGG. Column 5 and 6 are the main substrate and product of the reaction. And column 7 shows catalyzed enzymes of the reaction.
(XLSM)

Table S2 **Transport reactions.** Column 1 is the transport indexes assigned by us. Column 2 (path: slc2cps) indicates the path number that transport belongs to. All transports are considered as reversible (Column 3). Column 4 and 5 are transportable metabolite ID and its official name. Column 6 shows slcs that can transport the metabolite.
(XLSM)

Table S3 **Gene expression in organs.** Column 1 and 2 indicate gene ID and gene symbol. The other columns are the expression scores of the corresponding organs.
(XLSM)

Table S4 **Scores for predefined sub-networks in 17 organs.** Column 1 and 2 list the predefined sub-networks and their functional descriptions. Column 3 shows kegg pathways that each sub-network belongs to. Column 4 and 5 list involved reactions (indicated by KEGG ID) and enzymes for each sub-network. The remaining 17 columns are the calculated scores for each sub-network in 17 organs.
(XLSM)

Table S5 **Statistical significances of tissue-specific metabolism/transport clusters.** All clusters with more than 20 reactions are selected and listed according to organs. Column 2 lists the number of reactions involved in clusters. Column 3 is the calculated score of the cluster. Column 4 is the mean calculated score from 100 times permutations (randomly assign scores for cluster reactions and calculate a score for this cluster). Column 5 is the p-value obtained from t-test (column 3 v.s column 4).
(XLS)

Table S6 **All transportable processes involved in tissue-specific metabolism/transport clusters.** Column 1 and 2 are the metabolite name and KEGG ID. Column 3 indicates the organ where metabolite is predicted to be transported. Column 4 shows the class that this transport process belongs to (coupled (class 1) or isolate (class 2)). Column 5 and 6 are the highest expression score and the corresponding slc that mediates the metabolite.
(XLS)

Table S7 **Concentrations of Slc transportable metabolites in human blood, urine, CSF and saliva derived from Human Metabolic Database (HMDB).** Column 1 indicates metabolite ID. Column 2 and 3 shows the organ and corresponding concentration.
(XLS)

Table S8 **Exclusively predicted transports in adipose, liver and muscle.** Column 1 and 2 list the organ and exclusively predicted transportable metabolite that can be transported in the organ. Column 3 is the expression score mediated by slcs in that organ. And column 4 is the transport type.
(XLS)

Data S1 (ZIP)
