# Supplementary figures and images for: A Computational Approach to Estimate Interorgan Metabolic Transport in a Mammal
Source: PLoS One. 2014 Jun 27;9(6):e100963. doi: 10.1371/journal.pone.0100963 (PMC4074118; doi:10.1371/journal.pone.0100963)

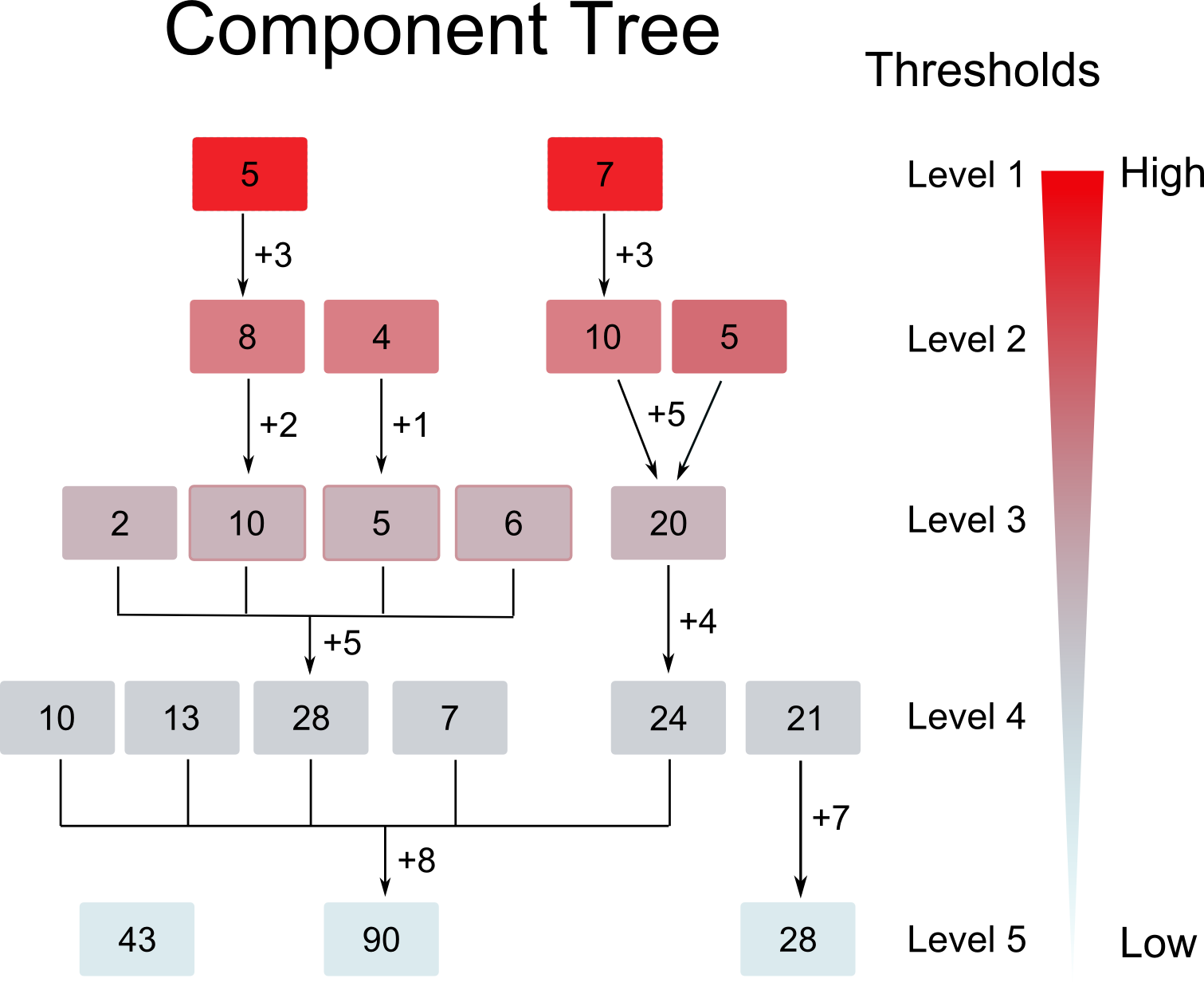

Supplement: Figure S1 — A schematic representation of the component tree. A component tree is a data structure representing the hierarchical relationship between components (rectangular boxes) obtained with the decreasing score threshold (see main Text). Components are connected nodes in the reference network with scores above a given threshold. The top of the component tree consists of the nodes with highest scores. At each step of decreasing threshold, new nodes are incorporated into the components and new components are generated. The numbers indicated on the components represent the numbers of nodes within the components. The numbers next to the arrows represent the numbers of new nodes added into the component as the threshold decreases. The colors of components ranging from red to light blue represent the decreasing threshold values of the scores. (PNG) [file pone.0100963.s001.png]

# Organ-Compound Interaction Matrix

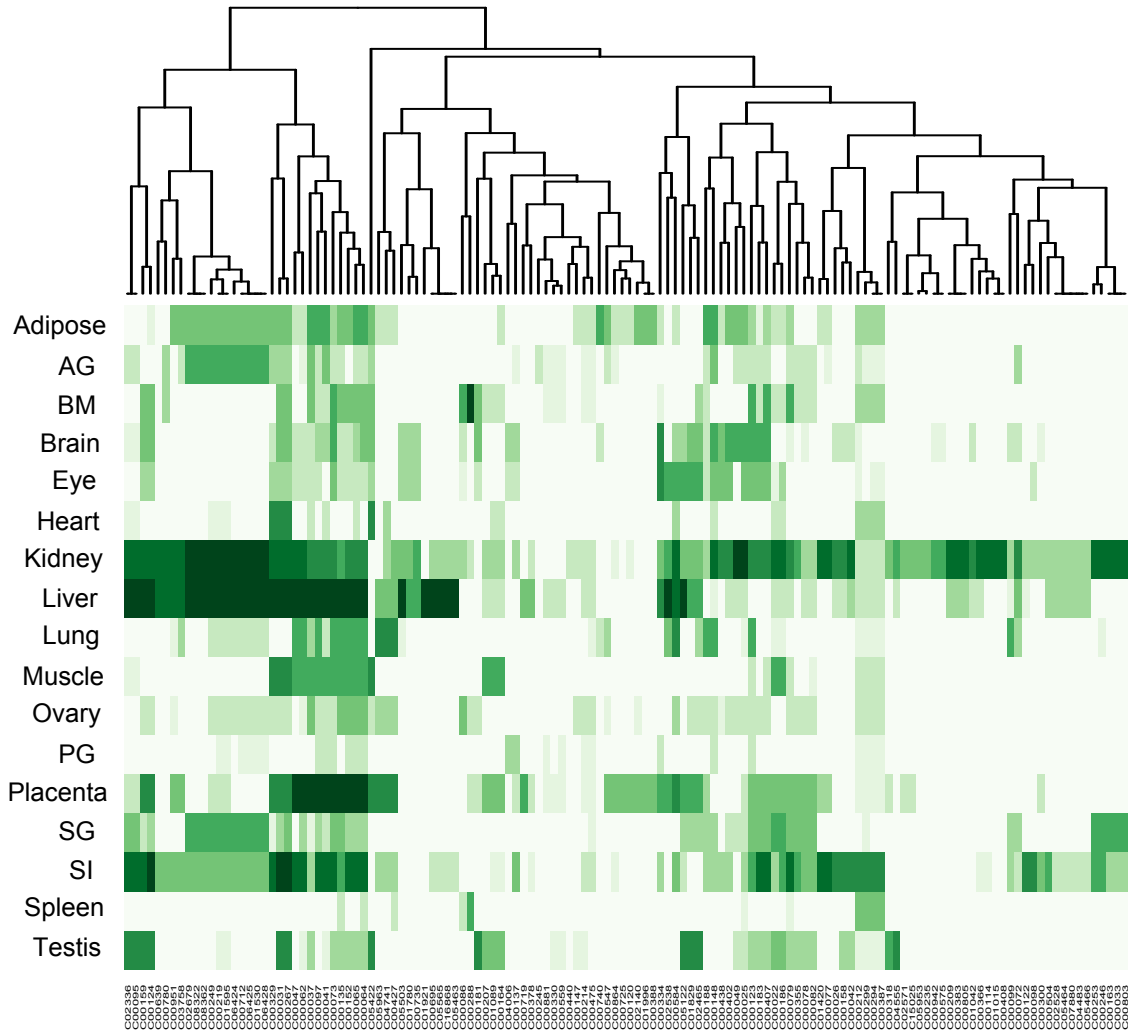

Supplement: Figure S4 — Organ transport capability for all transportable metabolites. Rows are 17 organs. Columns are 136 transport processes. Cell colors reflect high (dark green) to low (white) transport capabilities of corresponding metabolites for a given organ. (PDF) [file pone.0100963.s004.pdf]

A

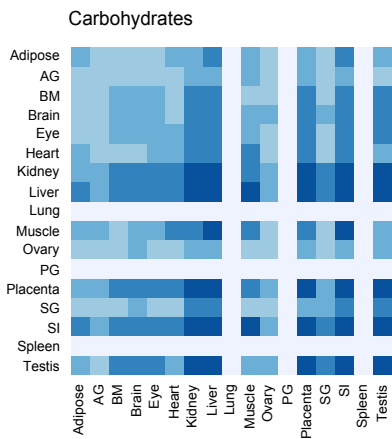

B

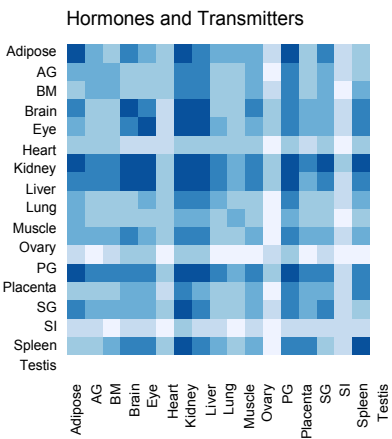

C

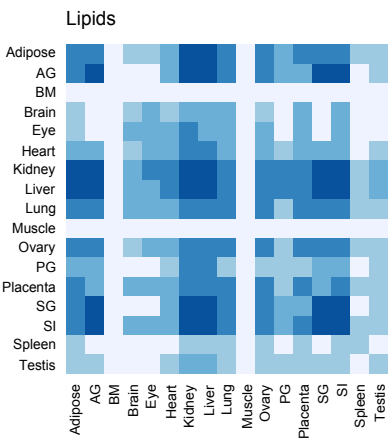

D

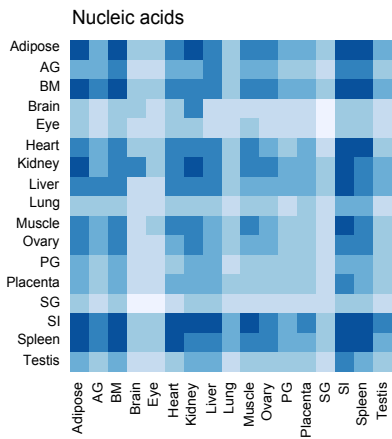

E

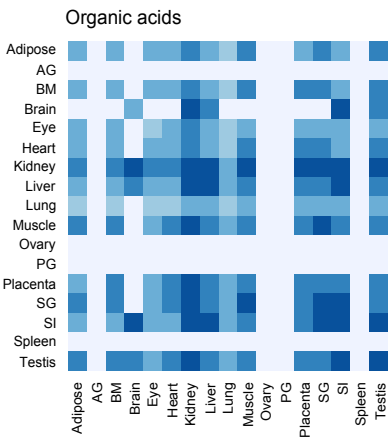

F

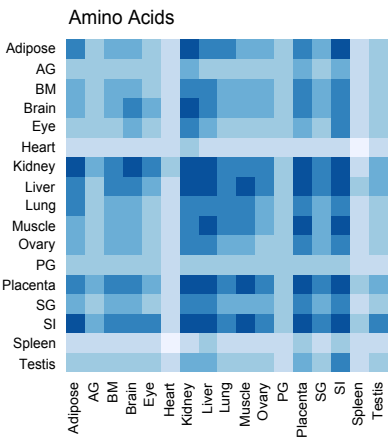

G

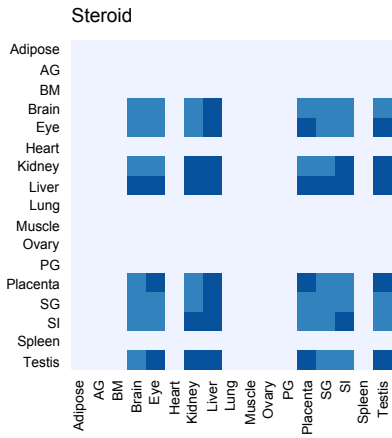

Supplement: Figure S5 — Organ-organ connections via specific transportable metabolites. 136 Slc-mediated metabolites are classified into seven categories: (A) Carbohydrates; (B) Hormones and transmitters; (C) Lipids; (D) Nucleic acids; (E) Organic acids; (F) Amino acids; (G) Steroid. Each heatmap matrix reflects organ-organ connection through a certain type of transportable metabolites. Cell colors reflect the degree of connectivity between organs. (PDF) [file pone.0100963.s005.pdf]

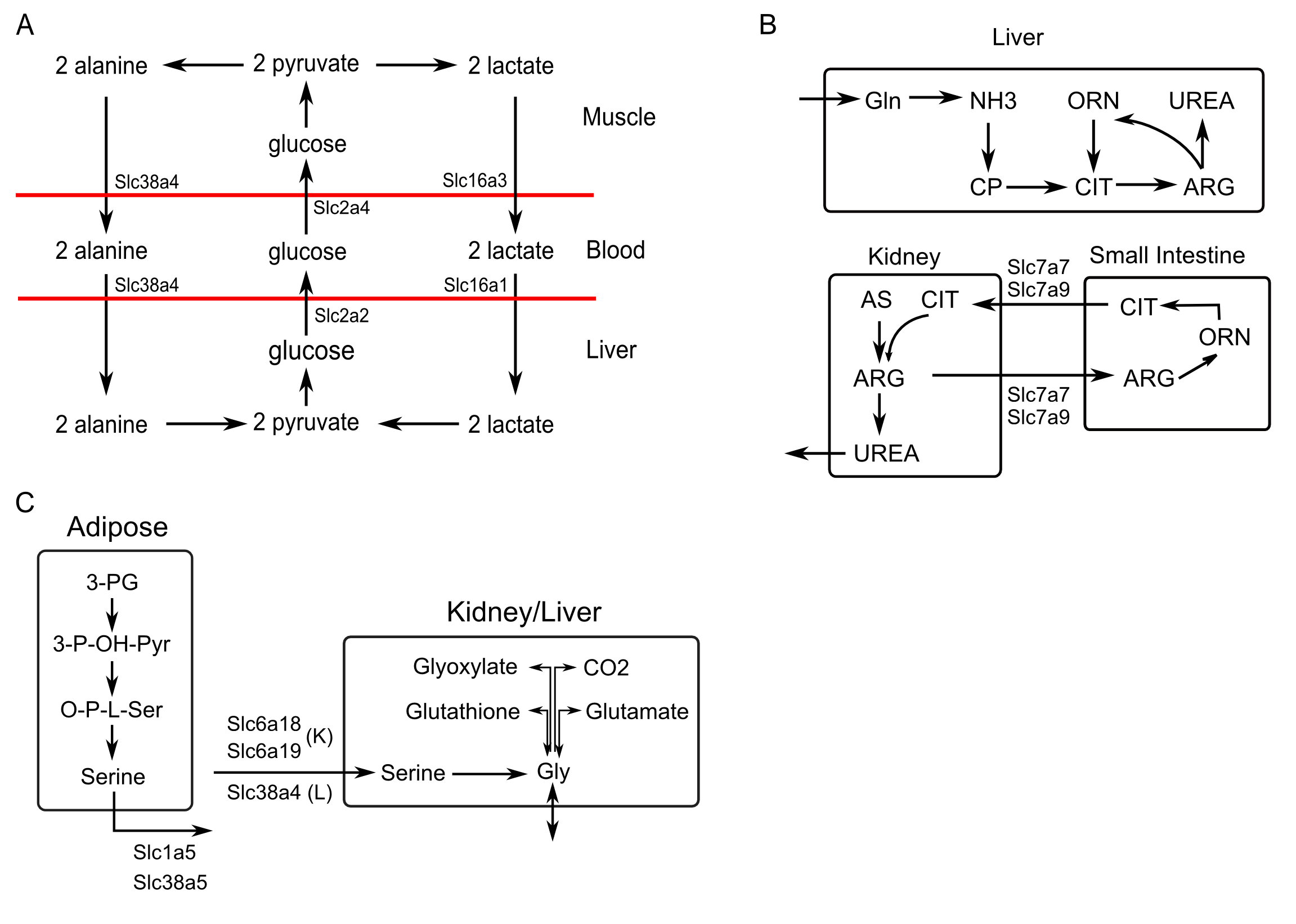

Supplement: Figure S6 — Selected examples of interorgan metabolism/transport processes and predicted transporters. (A) Cori Cycle and Glucose-Alanine Cycle between liver and muscle. (B) Urea Cycle is realized in full in liver but only partially in kidney and in small intestine. This leads to the transport of citrulline and arginine between small intestine and kidney in the so-called citrulline-arginine shunt. (C) Serine synthesis in adipose and serine-glycine conversion in liver and kidneys may lead to the transport of serine between adipose and liver and kidney. Gln, glutamine; ORN, ornithine; CP, carbamoyl phosphate; CIT, citrulline; ARG, arginine; AS, aspartate; 3-PG, 3-Phosphoglycerate; 3-P-OH-Pyr, 3-Phosphonooxypyruvate; O-P-L-Ser, 3-Phosphoserine; Gly, Glycine. (TIF) [file pone.0100963.s006.tif]

A

Bordbar's      Ours

Adipose

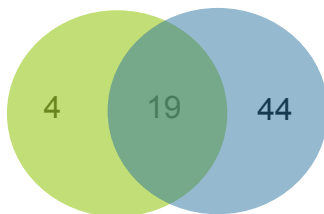

Liver

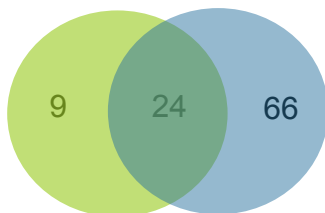

Muscle

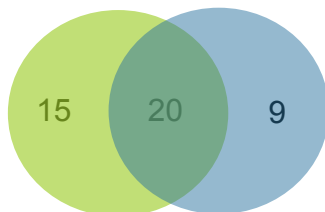

B

249 metabolites from Shlomi

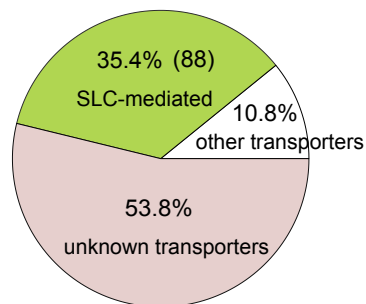

SLC-mediated metabolites

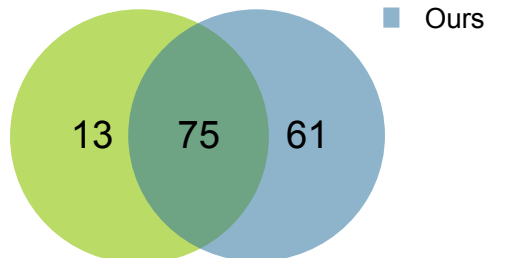

Supplement: Figure S7 — Comparison of predicted Slc-mediated transports. (A)Comparison of predicted Slc-mediated transports between Bordbar’s result [15] and ours in adipose, liver and muscle. (B) Comparison of predicted Slc-mediated transports between Shlomi et al’s result and ours in liver and kidney [8]. (PDF) [file pone.0100963.s007.pdf]
