# Supplementary material for: A Computational Approach to Estimate Interorgan Metabolic Transport in a Mammal
Source: PLoS One. 2014 Jun 27;9(6):e100963. doi: 10.1371/journal.pone.0100963 (PMC4074118; doi:10.1371/journal.pone.0100963)
Supplement: Figure S2 — Complete overview of Tissue-specific metabolism/transport clusters. This is a complete collection of tissue-specific metabolism/transport clusters of all 17 organs investigated in our study. The detailed information of these diagrams can be found in the legend of Figure 3. (PDF) [file pone.0100963.s002.pdf]

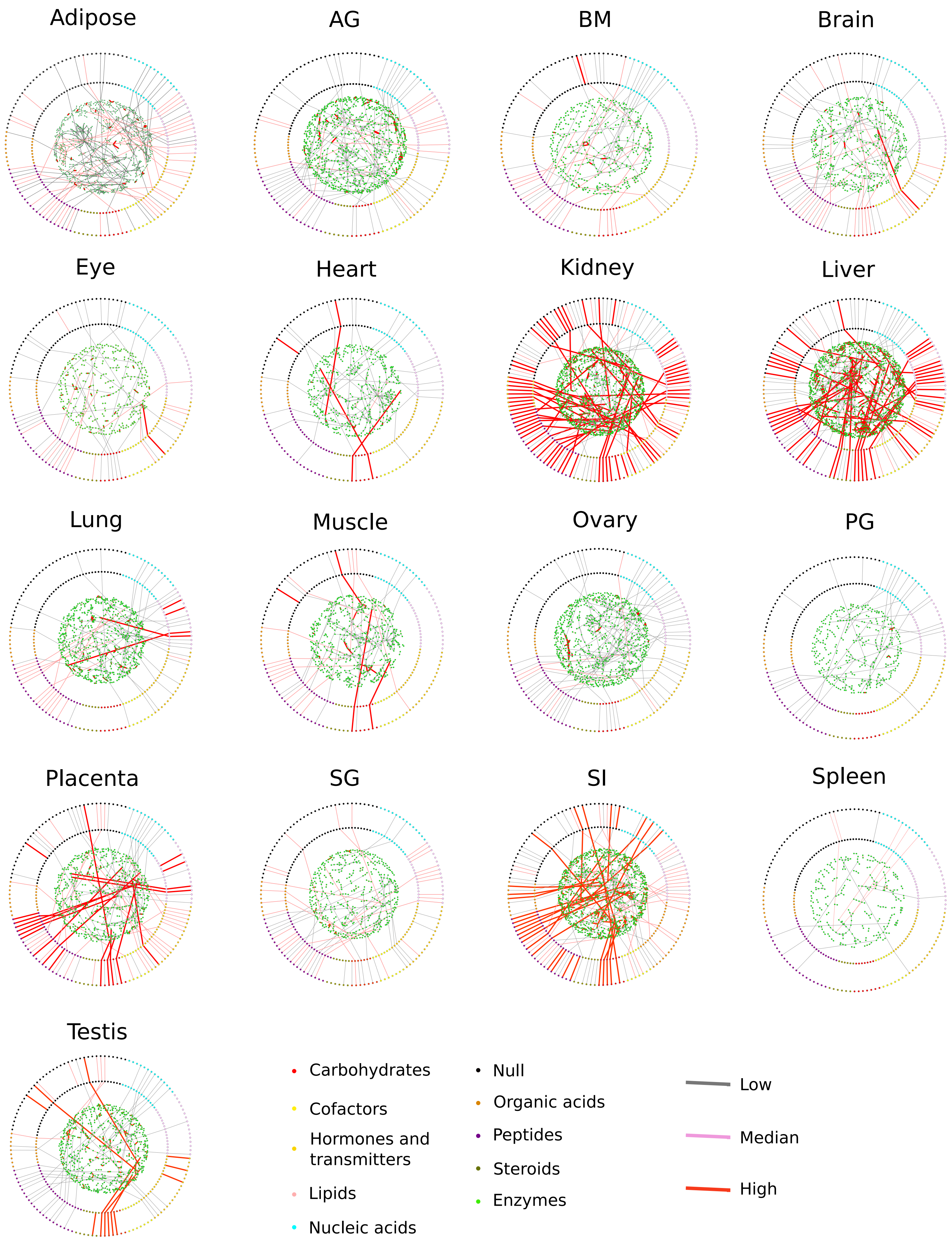

Adipose
AG
BM
Brain
Eye
Heart
Kidney
Liver
Lung
Muscle
Ovary
PG
Placenta
SG
SI
Spleen
Testis
Carbohydrates
Cofactors
Hormones and transmitters
Lipids
Nucleic acids
Null
Organic acids
Peptides
Steroids
Enzymes
Low
Median
High
