# Supplementary material for: A Computational Approach to Estimate Interorgan Metabolic Transport in a Mammal
Source: PLoS One. 2014 Jun 27;9(6):e100963. doi: 10.1371/journal.pone.0100963 (PMC4074118; doi:10.1371/journal.pone.0100963)
Supplement: Figure S3 — Three different types of expression patterns of reactions in metabolic pathways across organs. The metabolic reactions that are ubiquitous in at least 10 organs (A), restricted to 3–9 organs (B), or highly restricted to only one or two organs (C). The score of each reaction in the 17 organs is provided. The reactions are marked in red color for organs with high scores. The reactions in (A) and (B) belonging to different metabolic pathways form clusters (rectangular boxes with different colors) such as purine metabolism, pyrimidine metabolism and so on. (C) Heme biosynthesis pathway mainly occurs in bone marrow and hormones such as estrone can be synthesized by the ovary and adrenaline synthesis is restricted to the adrenal gland. The synthesis of niacin (vitamin B3) from tryptophan is restricted to liver. Calcidiol biosynthesis from vitamin D3 is specific for liver while its conversions into calcitriol and secalciferol are specific for kidney. (PDF) [file pone.0100963.s003.pdf]

A

Purine Metabolism

Reaction	Liver	Small Intestine	Kidney	Testis	Adrenal Gland	Salivary Gland	Lung	Ovary	Adipose	Placenta	Eye	Pituitary Gland	Brain	Spleen	Bone Marrow	Heart	Muscle
rn:R00434	0.663	5.059	0.792	4.892	2.077	2.52	3.669	1.925	1.937	3.164	2.675	1.849	3.781	2.329	1.834	1.125	2.492
rn:R01234	1.584	2.962	2.648	3.164	3.512	1.113	2.801	0.95	2.081	2.726	8.209	2.039	4.077	1.511	2.399	1.388	2.546
rn:R00441	0.922	1.134	1.645	1.89	1.694	0.407	0.42	1.326	0.792	1.604	0.641	1.272	1.21	0.756	1.156	0.81	1.585
rn:R00330	0.87	0.117	1.042	4.752	1.622	0.662	1.347	0.852	0.501	0.503	-0.04	1.281	0.471	0.145	0.754	1.104	1.114
rn:R00328	3.715	4.358	3.073	2.475	1.064	2.285	1.894	1.331	0.874	1.466	0.263	2.134	1.117	0.661	0.441	1.887	0.757
rn:R00722	0.87	0.117	1.042	4.752	1.622	0.662	1.347	0.852	0.501	0.503	-0.04	1.281	0.471	0.145	0.754	1.104	1.114
rn:R00961	3.715	4.358	3.073	2.475	1.064	2.285	1.894	1.331	0.874	1.466	0.263	2.134	1.117	0.661	0.441	1.887	0.757
rn:R00376	0.415	1.388	0.22	2.89	0.566	0.068	0.023	0.825	1.494	1.095	0.651	0.614	-0.11	2.076	3.329	0.301	0.559
rn:R01857	0.87	0.117	1.042	4.752	1.622	0.662	1.347	0.852	0.501	0.503	-0.04	1.281	0.471	0.145	0.754	1.104	1.114
rn:R01227	0.149	1.792	2.565	7.538	1.365	0.259	2.544	1.358	0.478	1.942	2.22	0.533	0.853	0.867	2.494	1.009	1.329
rn:R02719	0.149	1.792	2.565	7.538	1.365	0.259	2.544	1.358	0.478	1.942	2.22	0.533	0.853	0.867	2.494	1.009	1.329
rn:R01126	0.149	1.792	2.565	7.538	1.365	0.259	2.544	1.358	0.478	1.942	2.22	0.533	0.853	0.867	2.494	1.009	1.329
rn:R00181	0.032	0.544	0.068	0.147	2.369	0.275	0.512	1.284	0.264	2.084	0.659	1.406	1.177	0.036	1.309	0.99	8.491
rn:R00183	0.149	1.792	2.565	7.538	1.365	0.259	2.544	1.358	0.478	1.942	2.22	0.533	0.853	0.867	2.494	1.009	1.329
rn:R00127	1.955	2.199	4.399	4.023	1.283	0.272	2.988	2.383	1.643	1.282	0.593	0.838	4.87	-0.09	0.593	4.067	5.337
rn:R00086	3.715	4.358	0.387	2.887	0.247	2.285	1.894	1.331	2.091	2.699	0.263	2.134	1.117	0.661	0.441	0.721	0.622
rn:R00124	0.87	0.117	1.042	4.752	1.622	0.662	1.347	0.852	0.501	0.503	-0.04	1.281	0.471	0.145	0.754	1.104	1.114
rn:R00435	0.922	1.134	1.645	1.89	1.694	0.407	0.42	1.326	0.792	1.604	0.641	1.272	1.21	0.756	1.156	0.81	1.585
rn:R00191	1.584	0.667	2.648	3.164	3.512	1.113	2.801	0.95	2.081	2.726	2.055	2.039	4.077	1.511	2.399	1.388	2.546
rn:R00089	0.373	1.115	0.792	4.892	2.077	1.867	3.035	1.312	1.937	3.164	2.675	1.849	3.781	2.329	1.834	1.125	2.492
rn:R01137	0.87	0.117	1.042	4.752	1.622	0.662	1.347	0.852	0.501	0.503	-0.04	1.281	0.471	0.145	0.754	1.104	1.114
rn:R00375	0.415	1.388	0.22	2.89	0.566	0.068	0.023	0.825	1.494	1.095	0.651	0.614	-0.11	2.076	3.329	0.301	0.559
rn:R01547	1.955	2.199	4.399	4.023	1.283	0.272	2.988	2.383	1.643	1.282	0.593	0.838	4.87	-0.09	0.593	4.067	5.337
rn:R02088	0.149	1.792	2.565	7.538	1.365	0.259	2.544	1.358	0.478	1.942	2.22	0.533	0.853	0.867	2.494	1.009	1.329
rn:R00507	4.229	5.807	2.676	3.668	1.807	3.117	2.115	2.029	3.64	4.944	1.308	1.145	1.345	0.852	1.603	1.703	1.414

Pyrimidine Metabolism

Reaction	Liver	Small Intestine	Kidney	Testis	Adrenal Gland	Salivary Gland	Lung	Ovary	Adipose	Placenta	Eye	Pituitary Gland	Brain	Spleen	Bone Marrow	Heart	Muscle
rn:R00377	0.415	1.388	0.22	2.89	0.566	0.068	0.023	0.825	1.494	1.095	0.651	0.614	-0.11	2.076	3.329	0.301	0.559
rn:R02326	0.87	0.117	1.042	4.752	1.622	0.662	1.347	0.852	0.501	0.503	-0.04	1.281	0.471	0.145	0.754	1.104	1.114
rn:R01664	0.149	1.792	2.565	7.538	1.365	0.259	2.544	1.358	0.478	1.942	2.22	0.533	0.853	0.867	2.494	1.009	1.329
rn:R00378	0.415	1.388	0.22	2.89	0.566	0.068	0.023	0.825	1.494	1.095	0.651	0.614	-0.11	2.076	3.329	0.301	0.559
rn:R02093	0.87	0.117	1.042	4.752	1.622	0.662	1.347	0.852	0.501	0.503	-0.04	1.281	0.471	0.145	0.754	1.104	1.114
rn:R01569	0.149	1.792	2.565	7.538	1.365	0.259	2.544	1.358	0.478	1.942	2.22	0.533	0.853	0.867	2.494	1.009	1.329
rn:R00570	0.87	0.117	1.042	4.752	1.622	0.662	1.347	0.852	0.501	0.503	-0.04	1.281	0.471	0.145	0.754	1.104	1.114
rn:R00442	0.922	1.134	1.645	1.89	1.694	0.407	0.42	1.326	0.792	1.604	0.641	1.272	1.21	0.756	1.156	0.81	1.585
rn:R00443	0.922	1.134	1.645	1.89	1.694	0.407	0.42	1.326	0.792	1.604	0.641	1.272	1.21	0.756	1.156	0.81	1.585
rn:R00156	0.87	0.117	1.042	4.752	1.622	0.662	1.347	0.852	0.501	0.503	-0.04	1.281	0.471	0.145	0.754	1.104	1.114
rn:R00155	3.715	4.358	3.073	2.475	1.064	2.285	1.894	1.331	0.874	1.466	0.263	2.134	1.117	0.661	0.441	1.887	0.757
rn:R00963	0.149	1.792	2.565	7.538	1.365	0.259	2.544	1.358	0.478	1.942	2.22	0.533	0.853	0.867	2.494	1.009	1.329
rn:R00511	0.149	1.792	2.565	7.538	1.365	0.259	2.544	1.358	0.478	1.942	2.22	0.533	0.853	0.867	2.494	1.009	1.329

Glutathione Metabolsim

Reaction	Liver	Small Intestine	Kidney	Testis	Adrenal Gland	Salivary Gland	Lung	Ovary	Adipose	Placenta	Eye	Pituitary Gland	Brain	Spleen	Bone Marrow	Heart	Muscle
rn:R00494	2.287	7.108	8.037	1.223	0.497	0.699	0.115	0.848	2.584	3.458	0.762	2.004	2.402	0.46	0.133	0.132	0.058
rn:R01262	2.287	7.108	8.037	1.223	0.497	0.699	0.115	0.848	2.584	3.458	0.762	2.004	2.402	0.46	0.133	0.132	0.058
rn:R03522	5.714	6.882	6.015	7.165	2.983	2.245	3.739	2.996	2.714	0.853	2.779	0.56	1.621	0.625	2.537	2.051	1.867
rn:R03916	2.287	7.108	8.037	1.223	0.497	0.699	0.115	0.848	2.584	3.458	0.762	2.004	2.402	0.46	0.133	0.132	0.058
rn:R01528	3.39	2.103	2.513	0.51	2.505	1.622	2.276	2.87	2.554	2.335	0.365	0.406	-0.48	-0.01	1.813	3.342	1.049
rn:R00267	3.39	2.103	2.513	0.51	2.505	1.622	2.276	2.87	2.554	2.335	0.365	0.406	-0.48	-0.01	1.813	3.342	1.049
rn:R02736	3.39	2.103	2.513	0.51	2.505	1.622	2.276	2.87	2.554	2.335	0.365	0.406	-0.48	0.115	1.813	3.342	1.049
rn:R02824	1.607	6.803	4.709	3.833	2.867	0.794	2.701	3.159	3.838	3.083	0.161	1.611	1.53	0.558	1.54	1.335	0.801
rn:R03915	1.607	6.803	4.709	3.833	2.867	0.794	2.701	3.159	3.838	3.083	0.161	1.611	1.53	0.558	1.54	1.335	0.801
rn:R03167	1.607	6.803	4.709	3.833	2.867	0.794	2.701	3.159	3.838	3.083	0.161	1.611	1.53	0.558	1.54	1.335	0.801
rn:R00274	1.607	6.803	4.709	3.833	2.867	0.794	2.701	3.159	3.838	3.083	0.161	1.611	1.53	0.558	1.54	1.335	0.801

Glycolysis Pentose Phosphate

Reaction	Liver	Small Intestine	Kidney	Testis	Adrenal Gland	Salivary Gland	Lung	Ovary	Adipose	Placenta	Eye	Pituitary Gland	Brain	Spleen	Bone Marrow	Heart	Muscle
rn:R01786	2.566	4.842	0.282	2.412	0.456	0.949	0.339	0.248	1.403	1.97	2.347	1.536	1.079	0.781	2.696	2.631	2.802
rn:R09085	2.566	4.842	0.282	2.412	0.456	0.949	0.339	0.248	1.403	1.97	2.347	1.536	1.079	0.781	2.696	2.631	2.802
rn:R01600	2.566	4.842	0.282	2.412	0.456	0.949	0.339	-0.03	1.403	1.97	2.347	1.536	1.079	0.781	2.696	2.631	2.802
rn:R00703	0.286	0.996	2.961	9.805	0.352	0.759	0.497	1.148	0.58	0.596	0.312	-0.03	2.016	0.043	0.891	3.133	1.752
rn:R00710	2.733	5.788	2.922	1.813	1.44	1.906	2.298	1.401	1.427	4.554	5.357	1.251	-0.02	0.323	0.929	0.517	0.02
rn:R00711	2.733	5.788	2.922	1.813	1.44	1.906	2.298	1.401	1.427	4.554	5.357	1.251	-0.02	0.323	0.929	0.517	0.02
rn:R04779	-0.05	1.484	0.101	1.187	0.268	-0.29	-0.08	0.346	0.398	1.036	1.142	0.815	2.373	0.092	0.366	3.498	5.014
rn:R04780	8.423	6.118	8.571	7.858	0.959	2.812	0.707	0.035	0.97	2.05	-0.2	-0.05	-0.15	-0.17	0.028	3.202	4.359
rn:R01070	8.41	7.933	8.816	-0.37	1.133	0.006	0.963	0.908	0.431	0.97	2.432	1.991	4.244	-0.5	-0.39	1.244	2.432
rn:R02736	3.39	2.103	2.513	0.51	2.505	1.622	2.276	2.87	2.554	2.335	0.365	0.406	-0.48	0.115	1.813	3.342	1.049
rn:R02035	3.39	0.585	2.224	0.51	2.03	1.622	2.276	1.849	2.554	2.335	0.365	0.406	-0.5	0.115	1.813	-0.02	-0.16
rn:R01528	3.39	2.103	2.513	0.51	2.505	1.622	2.276	2.87	2.554	2.335	0.365	0.406	-0.48	-0.01	1.813	3.342	1.049

Glycerolipid Metabolism

Reaction	Liver	Small Intestine	Kidney	Testis	Adrenal Gland	Salivary Gland	Lung	Ovary	Adipose	Placenta	Eye	Pituitary Gland	Brain	Spleen	Bone Marrow	Heart	Muscle
rn:R01752	2.733	5.788	2.922	0.948	1.44	1.224	1.573	1.401	1.427	0.773	-0.24	0.829	-0.13	0.092	0.296	0.517	-0.1
rn:R01041	0.32	6.019	1.789	2.321	9.547	0.953	1.04	2.228	0.514	0.657	1.764	0.472	-0.02	0.651	-0.01	1.544	1.657
rn:R01036	0.32	6.019	1.789	2.321	9.547	0.953	1.04	2.228	0.514	0.657	1.764	0.472	-0.02	0.651	-0.01	1.544	1.657
rn:R02241	2.436	2.43	1.026	2.12	2.468	0.029	1.243	1.343	3.031	0.914	1.664	1.487	1.026	-0.02	0.426	1.034	0.895
rn:R02239	2.339	2.139	0.891	1.529	2.236	1.27	2.078	0.748	1.666	1.901	0.688	0.855	0.889	0.023	0.622	1.714	2.407
rn:R02240	0.314	2.135	0.735	2.209	1.065	1.887	1.677	0.574	0.657	0.531	2.934	1.44	3.996	2.115	1.049	0.356	0.019
rn:R02687	6.148	4.681	0.738	1.423	0.675	2.384	2.297	2.645	2.946	4.27	2.627	0.341	0.458	5.949	0.143	1.129	0.819
rn:R02250	6.148	4.681	3.089	1.423	2.398	2.384	2.297	2.645	4.25	4.27	2.627	0.341	0.458	5.949	0.143	4.417	2.796
rn:R01350	6.148	4.681	3.089	1.423	2.398	2.384	2.297	2.645	4.25	4.27	2.627	0.341	0.458	5.949	0.143	4.417	2.796

B

Fatty Acid Metabolism

Reaction	Liver	Small Intestine	Kidney	Testis	Adrenal Gland	Salivary Gland	Lung	Ovary	Adipose	Placenta	Eye	Pituitary Gland	Brain	Spleen	Bone Marrow	Heart	Muscle
rn:R03990	3.082	1.634	2.612	0.379	1.918	0.118	0.159	0.432	1.631	0.509	-0.11	-0.31	-0.04	0.422	0.174	2.659	1.508
rn:R04740	5.144	3.951	6.163	0.365	1.263	0.994	-0.05	0.677	2.602	0.776	-0.91	-0.23	-0.62	-0.49	-0.42	1.536	0.572
rn:R04739	5.144	3.951	6.163	0.365	1.622	0.994	-0.07	0.544	2.602	0.776	-0.91	-0.27	-0.93	-0.49	-0.42	2.087	0.687
rn:R03857	3.082	1.634	2.612	0.379	1.918	0.118	0.159	0.432	1.631	0.509	-0.11	-0.31	-0.04	0.422	0.174	2.659	1.508
rn:R04170	5.144	3.951	6.163	0.365	1.263	0.994	-0.05	0.677	2.602	0.776	-0.91	-0.23	-0.62	-0.49	-0.42	1.536	0.572
rn:R04741	5.144	3.951	6.163	0.365	1.622	0.994	-0.07	0.544	2.602	0.776	-0.91	-0.27	-0.93	-0.49	-0.42	2.087	0.687
rn:R04754	3.082	1.634	2.612	0.379	1.918	0.118	0.159	0.432	1.631	0.509	-0.11	-0.31	-0.04	0.422	0.174	2.659	1.508
rn:R04744	5.144	3.951	6.163	0.365	1.263	0.994	-0.05	0.677	2.602	0.776	-0.91	-0.23	-0.62	-0.49	-0.42	1.536	0.572
rn:R04743	5.144	3.951	6.163	0.365	1.622	0.994	-0.07	0.544	2.602	0.776	-0.91	-0.27	-0.93	-0.49	-0.42	2.087	0.687
rn:R03777	3.082	1.634	2.612	0.379	1.918	0.118	0.159	0.432	1.631	0.509	-0.11	-0.31	-0.04	0.422	0.174	2.659	1.508
rn:R04746	5.144	3.951	6.163	0.365	1.263	0.994	-0.05	0.677	2.602	0.776	-0.91	-0.23	-0.62	-0.49	-0.42	1.536	0.572
rn:R04745	5.144	3.951	6.163	0.365	1.622	0.994	-0.07	0.544	2.602	0.776	-0.91	-0.27	-0.93	-0.49	-0.42	2.087	0.687
rn:R04751	3.082	1.634	2.612	0.379	1.918	0.538	0.159	0.492	1.631	0.509	-0.11	0.01	-0.04	0.422	0.174	2.659	1.508
rn:R04749	5.144	3.951	6.163	0.365	1.263	0.994	-0.05	0.677	2.602	0.776	-0.91	-0.23	-0.62	-0.49	-0.42	1.536	0.572
rn:R04748	5.144	3.951	6.163	0.365	1.622	0.994	-0.07	0.544	2.602	0.776	-0.91	-0.27	-0.93	-0.49	-0.42	2.087	0.687
rn:R01175	3.082	1.634	2.612	0.379	1.918	0.538	-0.05	0.492	1.631	0.509	-0.11	0.01	-0.04	0.422	0.174	2.659	1.508
rn:R03026	5.144	3.951	6.163	0.365	1.263	0.994	-0.05	0.677	2.602	0.776	-0.91	-0.23	-0.62	-0.49	-0.42	1.536	0.572
rn:R01975	5.144	3.951	6.163	-1.34	1.622	0.994	-0.07	0.544	2.602	0.776	-0.91	-0.45	-0.93	-1.01	-1.03	2.087	0.687

Fatty Acid Elongation Biosynthesis

Reaction	Liver	Small Intestine	Kidney	Testis	Adrenal Gland	Salivary Gland	Lung	Ovary	Adipose	Placenta	Eye	Pituitary Gland	Brain	Spleen	Bone Marrow	Heart	Muscle
rn:R00391	2.57	0.399	1.088	-0.28	1.388	-0.16	0.18	0.726	0.696	-0.49	-0.76	-0.06	-1.05	-1.18	-1.45	2.545	1.287
rn:R01778	1.155	1.887	1.863	0.365	1.622	-0.56	-0.28	0.544	1.253	-0.11	-1.29	-0.27	-0.99	-0.49	-0.42	2.087	0.687
rn:R02685	1.523	0.384	2.007	1.651	0.972	-0.12	-0.05	0.893	2.982	0.31	-0.68	-0.23	-0.62	-0.49	-0.42	1.536	0.572
rn:R04386	1.024	-0.3	0	-0.04	1.818	0.656	0.256	1.703	1.88	-0.49	0.03	0.401	0.081	-0.32	-0.49	1.932	1.735
rn:R04385	1.024	-0.3	0	-0.04	1.818	0.656	0.256	1.703	1.88	-0.49	0.03	0.401	0.081	-0.32	-0.49	1.932	1.735
rn:R01626	1.104	-0.14	0.536	1.546	2.939	0.031	1.401	2.68	3.417	-0.26	-0.32	0.027	0.26	-0.15	-0.31	0.413	0.309
rn:R04355	1.381	0.274	1.346	0.581	2.939	0.031	1.401	2.68	3.417	0.011	-0.32	-0.11	0.26	-0.18	-0.1	0.697	0.443
rn:R04957	1.381	0.274	1.346	0.581	2.939	0.031	1.401	2.68	3.417	0.011	-0.32	-0.11	0.26	-0.18	-0.1	0.697	0.443
rn:R04726	1.381	0.274	1.346	0.581	2.939	0.031	1.401	2.68	3.417	0.011	-0.32	-0.11	0.26	-0.18	-0.1	0.697	0.443
rn:R04968	1.381	0.274	1.346	0.581	2.939	0.031	1.401	2.68	3.417	0.011	-0.32	-0.11	0.26	-0.18	-0.1	0.697	0.443

TCA Cycle

Reaction	Liver	Small Intestine	Kidney	Testis	Adrenal Gland	Salivary Gland	Lung	Ovary	Adipose	Placenta	Eye	Pituitary Gland	Brain	Spleen	Bone Marrow	Heart	Muscle
rn:R00014	0.45	0.437	1.476	6.979	0.78	0.555	-0.11	0.131	1.058	0.051	-0.07	-0.01	0.222	0.288	0.321	2.392	1.906
rn:R03270	1.972	1.494	2.203	6.979	1.635	1.584	0.162	0.508	1.98	0.051	-0.01	0.052	0.222	0.288	0.321	2.392	1.906
rn:R01899	2.5	2.103	2.513	-0.21	2.505	0.417	0.349	2.87	1.621	0.061	-0.34	-0.67	-0.48	-1.13	-0.18	3.342	1.049
rn:R00268	2.5	2.103	2.513	-0.21	2.505	0.417	0.349	2.87	1.621	0.061	-0.34	-0.67	-0.48	-1.13	-0.18	3.342	1.049
rn:R03316	1.972	1.494	2.203	6.979	1.635	1.584	0.162	0.508	1.98	0.051	-0.01	0.052	0.222	0.288	0.321	2.392	1.906
rn:R01082	1.677	0.682	1.468	-1.17	1.037	-0.45	-0.4	0.749	0.739	-0.45	-0.8	-0.3	-0.05	-0.36	-0.66	2.518	1.766

C

	Gene	Adipose	Adrenal Gland	Bone Marrow	Brain	Eye	Heart	Kidney	Liver	Lung	Muscle	Ovary	Pituitary Gland	Placenta	Salivary Gland	Small Intestine	Spleen	Testis
**Heme Biosynthesis**																		
Porphobilinogen																		
Hydroxymethylbilane	Hmbs	0.2	0.4	3.76	-1.2	-0.9	-0.1	0.18	0.85	-1.1	-0.3	0.44	-1	0.21	-0.1	-0	1.08	-0.6
Uroporphyrinogen III	Uros	0.01	-0.1	1.9	-0.2	-0.5	0.48	0.22	0.42	-0.7	0.45	-0.4	0.36	-0	-0.2	-0.5	0.03	-0.4
Coproporphyrinogen III	Urod	0.77	0.84	2.65	-0.7	-0.4	0.45	0.61	0.86	-0.5	0.73	0.34	-0.3	-0.7	-0.1	-0.7	-0	-1.4
Protoporphyrinogen IX	Cpox	-0.9	-0.7	2.59	-0.1	-0.3	0.53	1.35	1.88	-0.4	0.02	-0.2	-0.1	0.05	0.45	0.16	0.33	-2
Protoporphyrin IX	Ppox	0.11	0.56	2.29	-0.5	-0.5	-0.2	0.1	0.03	-0.1	-0.3	0.8	0.18	-0.5	0.07	-0.2	0.27	-1
Heme	Fech	0.16	0.73	2.66	-0.5	-1.1	1.15	1.05	1.41	-0.2	1.23	-0.1	-0.2	0.01	-0.9	-0.9	0.61	-1.3
**L-Dopa -> L-Adrenaline**																		
L-Dopa																		
Dopamine	Ddc	0.02	3.34	-0.9	1.12	-0.9	0.15	3.62	3.57	0.01	-0.7	-1.1	0.41	0.31	-0.9	4.69	-0.8	-0.9
L-Noradrenaline	Dbh	-0.1	5.65	0.03	-0.1	0.14	0.63	-0.1	0.02	-0.3	0.13	-0.3	-0.1	-0.01	0.13	-0.2	0.38	
L-Adrenaline	Pnmt	-0	5.57	-0.2	-0.1	0.39	0.1	-0.1	-0	-0.1	-0	0.26	0.35	-0.1	0.25	-0.2	0.08	0.32
**Tryptophan -> Niacin (Vitamin B3)**																		
Tryptophan																		
N-Formylkynurenine	Tdo2	5.12	0.87	0.13	1.65	0	0.1	0.17	8.6	1.52	0.24	-0	-0.1	1.22	0.08	3.94	0.12	0.14
L-Kynurenine	Afmid	1.82	0.91	0.95	1.11	-0.2	1.03	7.81	2.86	0.19	0.97	-0	-0.2	0.16	-0.1	3.95	0.36	0.08
3-Hydroxy-L-kynurenine	Kmo	0.41	-0.2	1.22	-0.2	-0.2	0.04	4.62	6.18	0.31	-0	-0.1	-0.1	-0.2	-0	0.16	2.65	-0.2
3-Hydroxyanthranilate	Kynu	-0	-0.1	0.1	-0.1	-0	0.05	0.36	4.95	0.21	-0.1	-0.1	-0	0	0.03	0.09	1.38	0
2,3-carboxymuconate	Haao	0.28	-0.2	1.17	-0.2	0.05	0.05	5.67	7.1	0.64	-0.1	-0.2	-0.1	-0.1	0.1	-0	1.99	-0
Quinolinate		3	3	3	3	3	3	3	3	3	3	3	3	3	3	3	3	3
Nicotinate D-ribonucleotide	Qprt	-0.1	-0.1	0.02	-0.2	-0.1	-0	2.59	3.37	0.18	-0	-0.1	0.21	0.13	0.15	-0	0.03	-0
Nicotinamide	Naprt1	0.1	0.1	0.43	-0.6	-0.4	0.48	1.63	3.58	-0	-0.2	0.27	-0.5	-0.4	0.85	3.77	-0.4	-0.5
**Vitamine D3 -> Calcidiol -> Calcitetrol/Secalciferol**																		
Vitamin D3																		
Calcidiol	Cyp2r1	0.51	-0.1	0.02	-0.1	-0.1	-0	0.03	3.55	-0.1	-0	-0	-0.1	0.32	0.08	0.04	0.32	1.43
Calcitriol	Cyp27b1	-0.1	-0	0.17	-0.1	-0	0.17	1.32	0.08	-0.1	0.01	-0	-0	0.04	0.18	-0	0.11	0.04
Calcitetrol	Cyp24a1	-0.1	-0	0.03	-0	0.04	0.14	4.49	0.01	-0	0.05	-0.1	0.01	0.45	0.05	0.07	-0	0.1
Secalciferol	Cyp24a1	-0.1	-0	0.03	-0	0.04	0.14	4.49	0.01	-0	0.05	-0.1	0.01	0.45	0.05	0.07	0	0.09
